# Supplementary material for: Postcranial osteology of Beipiaosaurus inexpectus (Theropoda: Therizinosauria)
Source: PLoS One. 2021 Sep 30;16(9):e0257913. doi: 10.1371/journal.pone.0257913 (PMC8483305; doi:10.1371/journal.pone.0257913)

PLOS ONE

Postcranial osteology of <i> Beipiaosaurus inexpectus </i>(Theropoda: Therizinosauria)

--Manuscript Draft--

**Manuscript Number:**	PONE-D-21-14221
**Article Type:**	Research Article
**Full Title:**	Postcranial osteology of <i> Beipiaosaurus inexpectus </i>(Theropoda: Therizinosauria)
**Short Title:**	Postcranial osteology of <i> Beipiaosaurus inexpectus </i>(Theropoda: Therizinosauria)
**Corresponding Author:**	Chun-Chi Liao<br>IVPP CAS: Institute of Vertebrate Paleontology and Paleoanthropology Chinese Academy of Sciences<br>Beijing, Beijing CHINA
**Keywords:**	anatomy, Early Cretaceous, Maniraptorans, morphology, Therizinosaurioid, Jehol Biota
**Abstract:**	<i> Beipiaosaurus inexpectus </i> , from the Lower Cretaceous Yixian Formation of Sihetun Locality near Beipiao, Liaoning, China, is a key taxon for understanding the early evolution of therizinosaurians. Since initial publication in 1999, only the cranial elements of this taxon have been described in detail. Here we present a detailed description of the postcranial skeletal anatomy of the holotype specimen of B. inexpectus , including two never before described dorsal vertebrae from the anterior half of the series. Based on these observations, and comparisons with the postcranial skeleton of therizinosaurian taxa named since the most recent diagnosis, we revised the diagnostic features for <i> B. inexpectus </i> adding three new possible autapomorphies (PII-3 shorter than PIII-4, subequal length of the pre- and postacetabular portions of the ilium, and equidimensional pubic peduncle of ilium). Additionally, we also propose three possible synapomorphies for more inclusive taxa (Therizinosauroidea and Therizinosauridae) and discuss implications for evolutionary trends within therizinosauria. The new acquired data from the postcranial osteology of the holotype specimen of <i> B. inexpectus </i> sheds new light on our understanding of postcranial skeletal evolution and identification of the therizinosaurians.
**Order of Authors:**	Chun-Chi Liao
	Lindsay E. Zanno
	Shiying Wang
	Xing Xu
**Additional Information:**	
**Question**	**Response**
**Financial Disclosure**<br><br>Enter a financial disclosure statement that describes the sources of funding for the work included in this submission. Review the submission guidelines for detailed requirements. View published research articles from *PLOS ONE* for specific examples.<br><br>This statement is required for submission and **will appear in the published article** if	This study was supported by the National Natural Science Foundation of China (41688103) and the scholarship provided by the University of Chinese Academy of Sciences (UCAS) to C-C L. The funders had no role in study design, data collection and analysis, decision to publish, or preparation of the manuscript.

Powered by Editorial Manager® and ProduXion Manager® from Aries Systems Corporation

the submission is accepted. Please make sure it is accurate.<br><br>**Unfunded studies**<br>Enter: *The author(s) received no specific funding for this work.*<br><br>**Funded studies**<br>Enter a statement with the following details:<br>• Initials of the authors who received each award<br>• Grant numbers awarded to each author<br>• The full name of each funder<br>• URL of each funder website<br>• Did the sponsors or funders play any role in the study design, data collection and analysis, decision to publish, or preparation of the manuscript?<br>• **NO** - Include this sentence at the end of your statement: *The funders had no role in study design, data collection and analysis, decision to publish, or preparation of the manuscript.*<br>• **YES** - Specify the role(s) played.<br><br>* typeset	
**Competing Interests**<br><br>Use the instructions below to enter a competing interest statement for this submission. On behalf of all authors, disclose any competing interests that could be perceived to bias this work—acknowledging all financial support and any other relevant financial or non-financial competing interests.<br><br>This statement is required for submission and **will appear in the published article** if the submission is accepted. Please make sure it is accurate and that any funding sources listed in your Funding Information later in the submission form are also declared in your Financial Disclosure statement.<br><br>View published research articles from *PLOS ONE* for specific examples.	The authors have declared that no competing interests exist.

**NO authors have competing interests**<br><br>Enter: *The authors have declared that no competing interests exist.*<br><br>**Authors with competing interests**<br><br>Enter competing interest details beginning with this statement:<br><br>*I have read the journal's policy and the authors of this manuscript have the following competing interests: [insert competing interests here]*<br><br>* typeset	
**Ethics Statement**<br><br>Enter an ethics statement for this submission. This statement is required if the study involved:<br><br>• Human participants<br>• Human specimens or tissue<br>• Vertebrate animals or cephalopods<br>• Vertebrate embryos or tissues<br>• Field research<br><br>Write "N/A" if the submission does not require an ethics statement.<br><br>General guidance is provided below. Consult the submission guidelines for detailed instructions. **Make sure that all information entered here is included in the Methods section of the manuscript.**	N/A

**Format for specific study types**<br><br>**Human Subject Research (involving human participants and/or tissue)**<br>• Give the name of the institutional review board or ethics committee that approved the study<br>• Include the approval number and/or a statement indicating approval of this research<br>• Indicate the form of consent obtained (written/oral) or the reason that consent was not obtained (e.g. the data were analyzed anonymously)<br><br>**Animal Research (involving vertebrate animals, embryos or tissues)**<br>• Provide the name of the Institutional Animal Care and Use Committee (IACUC) or other relevant ethics board that reviewed the study protocol, and indicate whether they approved this research or granted a formal waiver of ethical approval<br>• Include an approval number if one was obtained<br>• If the study involved *non-human primates*, add *additional details* about animal welfare and steps taken to ameliorate suffering<br>• If anesthesia, euthanasia, or any kind of animal sacrifice is part of the study, include briefly which substances and/or methods were applied<br><br>**Field Research**<br><br>Include the following details if this study involves the collection of plant, animal, or other materials from a natural setting:<br>• Field permit number<br>• Name of the institution or relevant body that granted permission	
**Data Availability**<br><br>Authors are required to make all data underlying the findings described fully available, without restriction, and from the time of publication. PLOS allows rare exceptions to address legal and ethical concerns. See the PLOS Data Policy and FAQ for detailed information.	Yes - all data are fully available without restriction

A Data Availability Statement describing where the data can be found is required at submission. Your answers to this question constitute the Data Availability Statement and **will be published in the article**, if accepted.<br><br>**Important:** Stating 'data available on request from the author' is not sufficient. If your data are only available upon request, select 'No' for the first question and explain your exceptional situation in the text box.<br><br>Do the authors confirm that all data underlying the findings described in their manuscript are fully available without restriction?	
**Describe where the data may be found in full sentences. If you are copying our sample text, replace any instances of XXX with the appropriate details.**<br><br>• If the data are **held or will be held in a public repository**, include URLs, accession numbers or DOIs. If this information will only be available after acceptance, indicate this by ticking the box below. For example: *All XXX files are available from the XXX database (accession number(s) XXX, XXX.).*<br>• If the data are all contained **within the manuscript and/or Supporting Information files**, enter the following: *All relevant data are within the manuscript and its Supporting Information files.*<br>• If neither of these applies but you are able to provide **details of access elsewhere**, with or without limitations, please do so. For example:<br><br>*Data cannot be shared publicly because of [XXX]. Data are available from the XXX Institutional Data Access / Ethics Committee (contact via XXX) for researchers who meet the criteria for access to confidential data.*<br><br>*The data underlying the results presented in the study are available from (include the name of the third party*	All relevant data are within the manuscript and its Supporting Information files.

and contact information or URL).<br>• This text is appropriate if the data are owned by a third party and authors do not have permission to share the data.<br><br>* typeset	
Additional data availability information:	Tick here if the URLs/accession numbers/DOIs will be available only after acceptance of the manuscript for publication so that we can ensure their inclusion before publication.

Powered by Editorial Manager® and ProduXion Manager® from Aries Systems Corporation

Manuscript
Click here to access/download;Manuscript;postcranial osteology of Beipiaosaurus inexpectus .docx

Postcranial osteology of *Beipiaosaurus inexpectus* (Theropoda: Therizinosauria)

Liao Chun-Chi [1, 2, 3], Lindsay E. Zanno[4, 5], Wang Shiying[1, 2, 3], Xu Xing [1, 2*]

[1] Key Laboratory of Vertebrate Evolution and Human Origins of Chinese Academy of Sciences, Institute of Vertebrate Paleontology and Paleoanthropology, Chinese Academy of Sciences, Beijing, China

[2] CAS Center for Excellence in Life and Paleoenvironment, Beijing, China

[3] University of Chinese Academy of Sciences, Beijing, China

[4] Paleontology, North Carolina Museum of Natural Sciences, Raleigh, North Carolina, USA

[5] Department of Biological Sciences, North Carolina State University, Raleigh, North Carolina, USA

* Corresponding author

E-mail: xu.xing@ivpp.ac.cn

Abstract

Beipiaosaurus inexpectus, from the Lower Cretaceous Yixian Formation of Sihetun Locality near Beipiao, Liaoning, China, is a key taxon for understanding the early evolution of therizinosaurians. Since initial publication in 1999, only the cranial elements of this taxon have been described in detail. Here we present a detailed description of the postcranial skeletal anatomy of the holotype specimen of *B. inexpectus*, including two never before described dorsal vertebrae from the anterior half of the series. Based on these observations, and comparisons with the postcranial skeleton of therizinosaurian taxa named since the most recent diagnosis, we revised the diagnostic features for *B. inexpectus* adding three new possible autapomorphies (PII-3 shorter than PIII-4, subequal length of the pre- and postacetabular portions of the ilium, and equidimensional pubic peduncle of ilium). Additionally, we also propose three possible synapomorphies for more inclusive taxa (Therizinosauroidea and Therizinosauridae) and discuss implications for evolutionary trends within therizinosauria. The new acquired data from the postcranial osteology of the holotype specimen of *B. inexpectus* sheds new light on our understanding of postcranial skeletal evolution and identification of the therizinosaurians.

Keywords anatomy, Early Cretaceous, Maniraptorans, morphology, Therizinosaurioid, Jehol Biota

Introduction

Therizinosaurus were a rare and bizarre clade of maniraptoran dinosaurs. Late-branching taxa possessed characteristic features such as a rostrally endentulous snout,

dentary with a lateral shelf, small tightly packed lanceolate teeth, diminutive skulls, elongate neck, extremely broad and opisthopubic pelvis, shortened tibia, and tetradactyl pes [1-3]. Skeletal remains have been discovered from Cretaceous strata of Asia and North America [4-8], although *Eshanosaurus deguchiianus* from the Lufeng Formation, Yunnan, China might represent an Early Jurassic therizinosaurian [9,10].

Beipiaosaurus inexpectus is an early-branching therizinosaurian from the Lower Cretaceous Yixian Formation in Liaoning, China. As one of the earliest-branching members of the clade, the discovery of this taxon was key for detangling the broader phylogenetic relationships of therizinosaurians. Particularly, because it lacks many features in late-branching members of the clade that are convergent with sauropodomorphan dinosaurs such as a tetradactyl pes and broad proximal first metatarsal as well as providing direct evidence of the presence of filamentous feathers in this clade [8]. Despite the significance of *B. inexpectus* for detangling early anatomical evolution in the clade, detailed morphological descriptions are restricted to the cranial elements, select elements of the appendicular skeleton, the "pygostyle," and unique type of feathers called elongated broad filamentous feathers (EBFFs) [8, 11-13]). A detailed description of the postcranial skeleton of the is needed to further understand this important taxon. Here we present such a description of the postcranial skeletal osteology of the holotype specimen (IVPP V11559) in order to fill in new anatomical information on this key taxon and shed new light on the evolution of the postcranial skeleton within Therizinosauria.

Methods

We follow the phylogenetic terminology and definitions of Zanno [3] for

Therizinosauria, Therizinosauroidea, and Therizinosauridae. In contrast to most paleontological literature, we identify the three tetanuran manual digits as II-III-IV following the ornithological literature and some recent paleontological studies [14, 15]. Photography of *B. inexpectus* was performed in the Institute of Vertebrate Paleontology and Paleoanthropology. In each figure, the matrix and neighbouring bones were recolored to highlight the bone in Adobe Photoshop CC 2018.

Institutional Abbreviations: IVPP, Institute of Vertebrate Paleontology and Paleoanthropology, Beijing, China.

Systematic Paleontology

Dinosauria Owen, 1842

Theropoda Marsh, 1881

Coelurosauria sensu Gauthier, 1986

Therizinosauria Russell, 1997

Therizinosauroidea Russel and Dong, 1993

***Beipiaosaurus inexpectus* Xu, Wang et Tang, 1999**

Holotype IVPP V11559, a partial, semi-articulated skeleton including some cranial elements and most of the postcranial elements. (Note: After *Beipiaosaurus inexpectus* was named in 1999, additional materials of the holotype [IVPP V11559] were collected from the type locality [11]. The holotype specimen thus comprises more skeletal elements than listed in the first description.)

Type locality and horizon Sihetun locality near Beipiao, Liaoning, China. Lower Cretaceous (Aptian) Yixian Formation [16].

Revised diagnosis.

A small therizinosaurian possessing the following autapomorphies among Therizinosauria (newly added features noted with an asterisk*): postorbital process of frontal large and abruptly transits from orbital rim [13]; parietal with a long and sharp anterior process [13]; ventral ramus of parietal squamosal process extremely long [13]; and external mandibular fenestra deep dorsoventrally and extremely posteriorly located [13]; four fused caudal dorsals [3]; pygostyle incorporating up to seven caudal vertebrae [3]; "rectangular buttress" on MCI expressed as a triangular flange [3]; elongate lateral articular surface on manual phalanx I-I [8]; manual ungual of digit II shorter than digit III*; subequal length of pre- and post-acetabular process of ilium*; equidimensional pubic peduncle of ilium (anteroposterior width subequal to mediolateral width)*; obturator process of ischium sinusoidal, with ventrally deflected distal portion [3]; ischial boot approximately twice anteroposterior depth of distal shaft [3]; low ridge on cranial femoral shaft extending proximally from medial condyle [3].

Description and Comparisons

IVPP V 11559 is considered a skeletally immature individual because of the lack of fusion in the non-cranial axial skeleton including cervical vertebrae and corresponding cervical ribs, dorsal, sacral, and most caudal vertebral centra and their corresponding neural spines (except for posterior caudals) [17, 18]. Postcranial skeletal

elements that variably fuse during ontogeny in theropods such as the ipsilateral scapula and coracoid, distal carpals II and III (known to at least partially fuse later in ontogeny in *Falcarius*; [19]), and the distal tarsals and tibia are also unfused.

Axial Skeleton

IVPP V11559 preserves four cervical, six dorsal, three sacral and 30 caudal vertebrae (Figs 1-3, Table 1). Most therizinosaurians except *Jianchangosaurus* and *Nanshiungosaurus brevispinus* are represented by incomplete presacral vertebral columns [3, 20, 21]. There are 10 cervicals, 12 dorsals and five sacrals in *Jianchangosauru*[illegible]20] and 11 cervicals, 10 dorsals and five sacrals in *Nanshiungosaurus brevispinus* [22], although a more recent study suggests that *Nanshiungosaurus brevispinus* (NIGP V4731) has six sacrals [3].

Table 1. Measurements in millimeters of vertebrae of *B. inexpectus* (IVPP V 11559)

**Cervical vertebra (fig.1)**	**Width, maximum**	**Length, maximum**
Posterior cervical 1	-	-
Posterior cervical 2	-	-
Posterior cervical 3	-	46.3
Posterior cervical 4	-	-
**Dorsal vertebra (fig.2)**	**Length, maximum**	**Hight, maximum**
Antero-middle dorsal (Fig. 2A)	44.0	47.2
Middle dorsal (Fig. 2B)	-	-
Posterior dorsal 1	44.9	-
Posterior dorsal 2	40.9	-
Posterior dorsal 3	37.8	-
Posterior dorsal 4	37.1	-

**Sacral vertebra (fig. 3)**		
Antepenultimate sacral	38.6	-
Penultimate sacral	36.7	-
Posteriormost sacral	32.2	-
**Anterior to middle caudal vertebra (figs 3, 4A)**		
1	31.8	-
2	31.9	62.4
3	-	-
4	-	-
5	-	-
6	-	-
7	34.2	39.8
8	33.1	40.1
9	33.1	39.7
10	33.6	37.6
11	33.0	-
**Posterior caudal vertebra (fig.4B)**		
Posterior caudal 1	-	-
Posterior caudal 2	27.1	23.5
Posterior caudal 3	26.5	25.3
**Posteriormost caudal vertebra (fig.4D)**		
Posteriormost caudal 1	17.7	12.6
Posteriormost caudal 2	16.6	13.7
Posteriormost caudal 3	15.4	12.1
Posteriormost caudal 4	14.3	10.9
Posteriormost caudal 5	12.2	10.9
Posteriormost caudal 6	10.8	8.6

Posteriormost caudal 7	8.9	9.5
Posteriormost caudal 8	5.5	7.1
Posteriormost caudal 9	6.3	5.6
Posteriormost caudal 10	-	4.4
Posteriormost caudal 11	-	-
Posteriormost caudal 12	3.0	-
Posteriormost caudal 13	2.1	3.6

Cervicals

Four incomplete cervical vertebrae are present, exposed in dorsal and lateral view (Figs 1A-B), and all of them are poorly preserved. Given that these cervical vertebrae are not elongate, they likely derive from the posterior cervical series. The total number of cervicals for *B. inexpectus* is unknown, but based on the closely related taxa *Beipiaosaurus sp.* (STM 31-1) [12] and *Jianchangosaurus* [20], we estimate around nine or 10. The later-branching therizinosaurians, *'Nanshiungosaurus' bohlini* [22] and *Neimongosaurus* [23]), have 11-12 and 14 cervicals, respectively. An elongate neck with more than 10 cervicals is also seen in closely related oviraptorosaurians such as *Caudipteryx*, *Citipati*, and *Khaan* [24, 25].

Figure 1. Cervical vertebrae of *B. inexpectus* (IVPP V 11559) in dorsal view; A. digitally enhanced photograph; B. interpretive line drawing. **Abbreviations: cn**, centrum; **epi**, epipophyses; **le**, ligamentum elastici; **ifpr**, infraprezygapophyseal lamina; **inpo**, interpostzygapophyseal lamina; **ns**, neural spine; **PCV1-3**, posterior cervical vertebrae 1-3; **poz**, postzygapophyses; **prz**, prezygapophyses. Scale bar equal 2 cm.

In dorsal view, the neural arch is wider than its corresponding centrum, and both the pre- and postzygapophyses are lateral to the centrum, as in other therizinosaurs [19,

23, 26]. The neural spines of cervicals are extremely low, anteroposteriorly short [8], and mediolaterally compressed with a straight dorsal border, in lateral view. Low and undeveloped cervical neural spines is a characteristic trait observed in many other therizinosaurians, such as *Nothronychus* [27], *Neimongosaurus* [23], *Jianchangosaurus* [20], and *Falcarius* [19]. The neural spine is centered on the neural arch, making an 'X'-shape in dorsal view.

The articular surfaces of the postzygapophyseal facets face ventrally and are rounded. The prezygapophyses are robust and bear oval-shaped articular surfaces, similar to *Jianchangosaurus* [20]. In dorsal view, there is an intraprezygapophyseal lamina between the prezygapophyses, which only reaches the posterior margin of the articular facets. Between the postzygapophysis, there is a web of bone connected the two parts called intrapostzygapophyseal lamina (sensu Wilson, 1999 [28]), which is seen in other therizinosaurians such as *Falcarius* and *Neimongosaurus*, and is present on a broad distribution of coelurosaurs including ornithomimids, dromaeosaurs, troodontids and oviraptorosaurs [19]. This lamina restricted the ligamentum elastici to a small fossa at the base of the neural spine (the interspinous fossa). As in *Falcarius* and *Jianchangosaurus*, there are prominent epipophyses above the postzygapophysis [19, 20].

The cervical centra of *B. inexpectus* are amphicoelous. Most therizinosaurians have amphicoelous cervical centra (e.g., *Falcarius,* Zanno [19]; *Martharaptor* [29]; *Jianchangosaurus* [20]; *Neimongosaurus* [23]; and *Nothronychus* [27]); or amphiplatyan centra (*Alxasaurus* [6]; and *'Nanshiungosaurus' bohlini,* [30]). The preserved third posterior cervical centrum is only slightly longer than the longest preserved posterior dorsal centra (3%). However, despite shortening of the cervical centra posteriorly, all cervical centra remain anteroposteriorlly longer than the

preserved dorsal centra in *B. inexpectus*, as in other therizinosaurs. In *Neimongosaurus*, the penultimate cervical centrum is 17% longer than the longest dorsal centrum [23], and it is 25% and 12.5% longer in larger and smaller individuals, respectively in *Alxasaurus* [6].

Ribs are not fused to posterior cervicals, an ontogenetically variable feature that might strictly reflect the early ontogenetic stage of *B. inexpectus*. This condition is also seen in *Jianchangosaurus* [20] and *Beipiaosaurus sp*. [12]. Cervical ribs are slender and long, longer than corresponding centra. Ribs elongation is shared with *B. sp*. [12] and *Jianchangosaurus* [20], but contrasts with *Neimongosaurus* [23] and *Alxasaurus* [6], which bear short and broad cervical ribs.

Dorsals

Six dorsals are preserved, four of them are preserved in articulation and exposed in lateral view (Fig. 2C). These vertebrae were figured in Xu et al., ([8], Fig. 1). The remaining two preserved dorsal vertebrae are preserved in anterolateral and lateral view (Figs 2A, B) and were discovered isolated along with additional materials of the holotype specimen and published in Xu et al. [11]. The four articulated dorsals are interpreted as deriving from the posterior portion of the series, and the isolated two dorsals are interpreted as deriving from the anterior to middle portion.

Figure 2. Photographs [illegible]rsal vertebrae of *B. inexpectus* (IVPP V 11559) A. antero-middle dorsal in lateral view; B. middle dorsal in anterolateral view; C. posterior dorsals in lateral view. **Abbreviations:** **cn**, centrum; **D. ld**, lateral depression of dorsals; **nc**, neural canal; **ns**, neural spine; **pd,** pneumatic depression; **PDV1-4,** posterior dorsal vertebrae 1-4; **podl**, postzygadiapophyseal lamina; **poz**, postzygapophyses; **prdl**, prezygadiapophyseal lamina; **prz**, prezygapophyses; **tp**, transverse process. Scale bars equal 2 cm.

Neural sutures appear to be at least partially fused in the more middle dorsals (Figs 2A, B). The one preserved in lateral view is interpreted as an antero-middle dorsal based on its lamination, the low neural spine, arched postzygapophyses, and the longer centrum (Figure 2A). The lamina and fossa system are developed, yielding pre- and postzygadiapophyseal laminae. The well-developed lamina and fossa system is also a feature can be seen in many therizinosaurians, including *Falcarius* [19], *Jianchangosaurus* [20], *Lingyuanosaurus* [21], *Shuzhousaurus* [30], *Alxasaurus* [6], *Neimongosaurus* [23], and *Nanshiungosaurus brevispinus* [22]. In lateral view, the centrum is sub-rectangular in shape, and the ventral border is concave slightly, making the articular facets relatively expanded dorsoventrally. There is a depression on the lateral surface, positioned under the transverse process. The same feature can also be seen in the posterior dorsals.

The vertebra preserved in anterolateral view is interpreted as a middle dorsal based on the height of the neural spine and upswept angle of the transverse processes (Fig. 2B). The shape of the neural spine is gracile, and its dorsal surface is slightly expanded anteroposteriorly. In comparison with the antero-middle dorsal, the neural spine of this vertebra is dorsoventrally taller and approximately the same dorsoventral heightas the centrum. In this respect, the neural spine of *B. inexpectus* is more similar to that of later-branching therizinosaurians such as *Nothronychus* [31], *Alxasaurus* [6], '*Nanshiungosaurus*' *bohlini* [22], *Neimongosaurus* [23], and *Erliansaurus* [32], which possess relatively taller neural spines. Whereas in other earlier-branching members such as *Falcarius* [19], *Jianchangosaurus* [20], and *Lingyuanosaurus* [21], dorsal neural spines are robust, anteroposteriorly broaden, and reduced in height. The neural canal in anterior view is pentagon-shaped and dorsoventrally tall. Parapophyses are not

prominent, and are present as a depression on the anterior rim, flush with neural arch. In anterior view, the articular facets of the centra are oval in shape and dorsoventrally high. Articular facets are slightly concave, suggesting an amphicoelus condition.

Neural sutures are unfused in the preserved posterior dorsals (Fig. 2C), and may also be incompletely fused in the more anterior dorsals (Fig. 2A, B). The neural arches of the four articulated dorsals are crushed and the neural arch is exposed only in ventral view. The transverse processes are elongate (i.e., the mediolateral length of the transverse process is approximately equal to the dorsoventral height of the corresponding centrum. In ventral view, the transverse process narrows in mid-shaft and slightly expands anteroposteriorly at the distalmost point. Parapophyses are not prominent, and present as a depression on the anterior rim, flush with neural arch. Posterior dorsal centra are spool-shaped generally and subrectangular in lateral view, as in *Falcarius* [19] and *Neimongosaurus* [23]. Centra are apneumatic, but do bear a lateral depression.

Ribs of dorsals are incomplete. However, it can be observed that they are elongate, extremely slender, and subtriangular in cross-section (Fig. 4C).

Sacrals

The total number of sacrals in *B. inexpectus* is currently unknown. [illegible]st three sacrals are preserved in the materials found from the re-excavation. The antepenultimate and the penultimate sacrals are fused. However, the posteriormost centrum is separated. All of the sacral central are preserved in ventral view with only the dextral sacral ribs attached (Fig. 3). The centra of the last three sacrals are subrectangle in lateral view, unlike the sacrals of *Falcarius* which are strongly medially constricted producing an hourglass-like shape in ventral view [19]. The sacral ribs of

the three posteriormost sacrals are oriented laterally and expand anteroposteriorly. They are robust and their length is subequal to the width of centra.

Figure 3. Photographs of sacral and anterior caudal vertebrae of *B. inexpectus* (IVPP V 11559) **Abbreviations: cn,** centrum; **cdv1-2,** caudal vertebrate 1-2; **ns,** neural spine; **sv**, sacral vertebrate; **sr**, sacral rib. Scale bars equal 2 cm.

Caudals

The majority of caudal vertebrae were recovered among the second set of materials published in 2003 (Figs 3, 4A-B, 4D-E). These constitute a nearly complete tail with 30 caudals [11]. However, some caudal vertebrae are not preserved, thus the total number of caudals of *B. inexpectus* was more than 30. In early-branching therizinosaurians, *Falcarius* is estimated to have about 35 [19]. In later-branching therizinosaurians, such as *Neimongosaurus* [23] and *Alxasaurus* [6] an incomplete series of 22 and 21 caudals are preserved, respectively.

Most caudal vertebrae are transversely compressed and exposed in lateral view. All of the caudal centra are apneumatic, amphicoelous, and constricted medially and ventrally. The anterior caudal centra are relatively robust with a subequal height to length ratio. Caudal centra gradually become anteroposteriorly elongate, as in other early-branching therizinosaurians (e.g., *Falcarius* [19]), as well as theropods generally. This condition is different from that observed in late-branching therizinosaurians, such as *Alxasaurus* [6] and *Northychus* [31], which possess anteroposteriorly abbreviated distal caudals. Unlike in other portions of the vertebral column, the neural arches and centra are fused in the caudals.

Figure 4. Photographs of axial skeleton elements of *B. inexpectus* (IVPP V 11559) A. middle caudals in lateral view; B. posterior caudals in lateral view; C. proximal end of dorsal rib in lateral view; D.

posterior caudals with pygostyle-like structure; E. close-up for pygostyle-like structure. **Abbreviations: cn,** centrum; **cav3-12**, caudal vertebrate 3-11; **ch**, chevron; **cp**, capitulum; **ft**, fusion tubercle; **ns,** neural spine; **pcdv1-3**, posterior caudal vertebrae 1-3; **pmcdv 1-13**, posteriormost caudal vertebrae 1-13; **poz**, postzygapophyses; **prz**, prezygapophyses; **tb**, tuberculum; **tp**, transverse process. Scale bars equal 2 cm.

Neural spines in the anterior portions of the caudal series are subequal to the height of centra and reduce in height posteriorly. On the preserved first through third neural spines, the neural spines are less anteroposteriorly expanded, slightly posterodorsally oriented, and possess a convex dorsal margin. The neural spines become more box-shaped in lateral view and also shorten in anteroposterior length, maintaining their relative proportions. Neural spines of mid-caudal vertebrae are more posteriorly positioned, becoming flush with the postzygapophysis (Fig. 4A). Further posteriorly, neural spines are absent (Fig. 4B).

The pre- and postzygapophysis are steeply elevated and posteriorly inclined, as in most theropods generally, but more prominently in therizinosaurians and oviraptorosaurians [3]. In anterior caudal vertebrae, the prezygapophyses are more robust with oval articulation facets, and gradually become slenderer and tapering distally. Prezygapophyses are elongate, approximately half the length of the neural arch and longer than the postzygapophysis. Postzygapophysis are most crushed or not visible in anterior to mid-caudal vertebrae. In posterior vertebrae, the postzygapophyses remain relatively short. The transverse processes are mostly crushed or incomplete. When present it is clear they reduce from anteroposteriorly elongate and dorsoventrally thin processes to only a small tubercle in the base of the middle neural arches along the axis. Posteriorly, the transverse processes become more anteriorly positioned.

The five posteriormost caudal vertebrae are fused together in a pygostyle-like

structure (Figs 4D-E) [11], with ventrally concave and dorsally convex margins, as well as a blunt posterior end. A prominent line of fusion is still visible between the anterior two centra on the "pygostyle," whereas the last two caudal vertebrae are completely fused, and can only be distinguished by a well-developed tubercle (Fig. 4E). The neural spines of these five caudals are fused, and the margins are obscured. The phylogenetic distribution of this feature in other therizinosaurians is currently unknown, because the posteriormost portion of the caudals series is not definitively preserved in other therizinosaurians. When compared to the pygostyle-like structures exhibited by oviraptorosaurs, the "pygostyle" of *B. inexpectus* appears morphologically more similar to that of birds, in being more co-ossified and possessing stronger dorsally curved axes [11, 33].

Chevrons

Some chevrons are preserved in articulation with the caudals; they are approximately the same length as the centra with which they once articulated (Fig. 4A-B). The proximal articular facets are concave in dorsal view, with a more extended posterior process. The shafts of the chevrons are straight as in *Neimongosaurus* [23], but different from the morphology of *Falcarius* and *Alxasaurus* [19].

Pectoral Girdle

IVPP V11559 preserves the right scapula, both coracoids, and a partial furcula with its corresponding impression (Fig. 5, Table 2).

Table 2. Measurements in millimeters of pectoral girdle and forelimb elements of *B. inexpectus*

(IVPP V 11559)

Element	Length	Width	Note
Scapula, right	>194.5※	21.2*	※losing distalmost part and some part of blade in proximal end is broken<br>*Narrowest part
Coracoid	-	-	
Furcula	84.8	-	
Humerus, right	>163.9※	47.0*	※Distal end is broken, some midshaft might lost.<br>*Width is measured from humeral head to lateral side
Ulna, right	-	13.4	
Radius, right	>120.5※	19.2	※losing some midshaft
Distal carpal II	5.8	20.0	
Distal carpal III, left	15.8	15.0	
MCII, right	38.9	16.1	
MCIII, right	77.3	13.7	
MCIV, right	75.9	-	
PII-1, right	63.6		
PII-2, right	71.7		
PIII-1, right	50.9		
PIII-2, right	60.1		
PIII-3, right	86.1		
PIV-1	-		
PIV-2	-		
PIV-3, right	38.5		
PIV-4	-		

Furcula

A partial right epicleidium of the furcula and its exquisite impression are preserved. Together, these demonstrate that it is a widely arched bone with an oblate-shaped cross-section (Fig. 5B) [8]. Although in Xu et al. [8] noted the absence of the hypocleidium on the furcula of *B. inexpectus*, Zanno [19] suggested that the incomplete impression of the furcula and poor development of this feature in other taxa (i.e., *Falcarius*, [2]; *Nothronychus* [31]), makes this absence uncertain. In therizinosaurians, development of hypocleidium only preserved in early-branching *Neimongosaurus* lacks this feature entirely [19, 23].

The intraclavicular angle of *B. inexpectus* is ~~145 degrees, similar to that of *Neimongosaurus*, which is about 135 degrees [23, 31], and unlike that of *Falcarius*, which is more acute (~104 degrees [2]). The epicleidea of *B. inexpectus* is straight, not bowed at its termini. In general shape, the furcula of *B. inexpectus* most closely resembles that of *Neimongosaurus* [23], as opposed to *Nothronychus* and *Falcarius*, which display at least some lateral deflection of the epicleidaltermini [19, 31]. The distal termini of *B. inexpectus* tapers to a point, asin *Jianchangosaurus*, and unlike in *Neimongosaurus* which bears a robust epicleidea with blunt distal ends [23]. In general, the furculae of therizinosaurians including *B. inexpectus*, *Falcarius*, *Nothronychus*, and *Neimongosaurus* are gracile and V-shaped.

Figure 5. Photographs of pectoral girdle elements of *B. inexpectus* (IVPP V 11559) A. coracoids; B. furcula; C. scapula. **Abbreviations: bl**, blade; **cf**, coracoid foramen; **C. gf**, glenoid fossa of coracoid; **ct**, coracoid tubercle; **epi**, epicleidium; **S. gf**, Glenoid fossa of scapula; **S. d**, depression of scapula. Scale bars equal 2 cm.

Coracoid

Both coracoids are preserved, yet incomplete. The right element is more complete than the left element (Fig. 5A). The coracoids of *B. inexpectus* are subrectangular, which is a feature also seen in most other therizinosaurians and some maniraptoran theropods [8], except the coracoids of *Jianchangosaurus*, which are more similar to that of ornithomimosaurs in having a semicircular shape [20]. The anterior region is anteroposteriorly shortened and elongated dorsoventrally as in *Falcarius* [2], and unlike *Neimongosaurus*, in which the anterior portion is plate-like and deflected posteromedially [23]. The curvature is relatively continuous as in *Falcarius* [2], although not as strongly recurved, and unlike the hinge form in *Neimongosaurus* [23]. There is a pronounced, nearly crest-shaped coracoid tubercle [9] similar in form to *Falcarius* [19], that is best-preserved on the left coracoid, unlike the moderately developed coracoid tubercle in *Alxasaurus* [6]. As in *Alxasaurus* and *Falcarius*, the coracoid tubercle occurs close to the ventral margin of glenoid [2, 6]. The coracoid comprises slightly more than half of the glenoid. This condition is intermediate between that of the early-branching *Falcarius* [2], in which the scapular portion is greater, and *Neimongosaurus* in which the coracoid comprises about two-thirds of the glenoid [23]. Dorsal to the glenoid, there is a coracoid foramen, similar to that of *Falcarius* morphologically [2].

Scapula

A partial right scapula is preserved in medial view. There is damage to the proximal and distal ends (Fig. 5C). The scapula and coracoid are not fused, which is a feature present in early-branching therizinosaurians, including *Falcarius* [2], *Jianchangosaurus* [20], *Martharaptor* [29], as well as some later-branching taxa, such

as *Nothronychus* [27] and *Erliansaurus* [32]. In other late-branching therizinosaurians, including *Suzhousaurus* [30], *Neimongosaurus* [23], *Segnosaurus* [4] and *Therizinosaurus*, the scapula and coracoid are fused. However, since IVPP V 11559 is a skeletally immature individual, the unfused condition might be the result of early ontogenetic stage. The blade is long and slender, with a slight distal expansion. Expansion of the distal blade can also be observed in *Falcarius* [2], *Alxasaurus* [6], *Neimongosaurus* [23] and *Erliansaurus* [32]. The reverse condition, scapula blade tapering of the distal end, is noted for later-branching therizinosaurians, such as *Nothronychus* and *Segnosaurus* [2, 4, 26].

The cross-section of the proximal end of the blade is flat and thin, gradually decreasing in dorsoventral height. There is a depression that begins midway along the medial surface of the scapular blade on *B. inexpectus*. This feature may be enhanced by or entirely the result of crushing. Such a depression is absent on *Falcarius*, *Neimongosaurus*, and *Erliansaurus* (LZ pers obs.). The cross-section of the proximal blade is subtriangular, as in *Falcarius* [2]; with the transversely wider ventral base formed by a ridge in the medial surface. The glenoid fossa is a kidney-shaped facet that spans both the scapula and coracoid. It is oriented posteroventrally, similar to early-branching therizinosaurians such as *Falcarius* [2] and *Jianchangosaurus* [20], and unlike the glenoid of the relatively late-branching *Neimongosaurus* and *Nothronychus*, which faces more laterally [23, 26].

Forelimb

Both forelimbs are preserved in IVPP V11559 and are relatively complete (Figs 6-7, Table 2). Forelimbs are relatively longer than the short and stout hind limbs in *B. inexpectus*, a feature shared with many dromaeosaurids and early-branching avialans

[8, 34]. This feature is also present in some other early-branching to intermediate therizinosaurians. For instance, the ratio of humerus to femur in other therizinosaurian dinosaurs is as follows: *Jianchangosaurus* 76.7%, *Alxasaurus* 67.6%, *Erlianosaurus* 66.7%, *Neimongosaurus* 60.7%, and *Nothronychus* 38.7% [6, 20, 23, 27, 32]. Although the humerus of *B. inexpectus* is incompletely preserved, it is at least more than 60% of the femur length (Table 2-3).

Humerus

Both humeri are preserved. The right one is complete and preserved in anterior view; however, its proximal and distal ends are separated (Figs 6A-B). Only the distal part of the left humerus, preserved in posterior view, remains (Fig. 7B). The humerus is shorter than the scapula, and relatively straight. In early-branching therizinosaurians, (e.g., *Falcarius),* the humerus is slightly sigmoidal, more similar to that of oviraptorids and *Deinonychus* [2]. Whereas, in late-branching therizinosaurians, the humerus varies from relatively straight [5, 26] to strongly sigmoidal [2, 4].

Figure 6. Photographs of right forelimb elements of *B. inexpectus* (IVPP V 11559) A. humerus in anterior view; B. radius and ulna; C. humerus distal end with radius; D. carpus and manus elements. **Abbreviations: dc III-IV**, distal carpal III-IV; **ec**, ectepicondyle; **hh**, humeral head; **im,** intermedium; **int**, internal tuberosity; **MCII-IV**, metacarpus **II-IV**; **PII-1 to PIV-4**, phalanges II-1 to IV-4; **ra**, radiale; **r.r**, right radius; **r.u**, right ulna; **rc**, radial condyles. Scale bars equal 2 cm.

There is a pointed internal tuberosity in the proximal end of the humerus, with a depression to separate the tuberosity and humeral head, which is also present in most therizinosaurians and *Mononykus* [8, 35]. This depression extends across the posterior

aspect to the proximal surface [8], as in *Neimongosaurus*, *Segnosaurus* and *Erlikosaurus*, [2]. However, we note that poor preservation of the humerus suggests caution in interpreting the morphology of the internal tuberosity, humeral head, and depression separating these features. In most therizinosaurians, humeral head is hypertrophied both posteriorly and dorsally, appearing bulbous in form [2], whereas in IVPP V11559, only the dorsal extension of humeral head can only be confirmed due to the poor preservation. Only the base of the deltopectoral crest is preserved. It extended from the humeral shaft 90 degrees. By observing the broken scar, the deltopectoral crest should be about one-third of the humeral length, similar to the ratio of *Falcarius*, *Jianchangosaurus*, *Neimongosaurus* and *Erliansaurus* [2, 20, 23, 32], andshorter in form then *Segnosaurus* and *Therizinosaurus* [4, 5].

Figure 7. Photographs of left forelimb elements of *B. inexpectus* (IVPP V 11559) A. manus elements in lateral view; B. humerus distal end in posterior view; C. carpus and metacarpus; D. radius and ulna; E. manus elements in anterior view. **Abbreviations: ec**, ectepicondyle; **dcII-IV**, distal carpal II-IV; **enc**, entepicondyle; **l.r**, left radius; **l.u**, left ulna; **im,** intermedium; **MCII-III**, metacarpus **II-III**; **PII-1 to PIV-4**, phalanges II-1 to IV-4 **pt**, posterior tuberosity; **ra**, radiale; **rc**, radial condyles; **uc**, ulnar condyles. Scale bars equal 2 cm.

As in other early-branching therizinosaurians, the proximal and distal ends of humerus are moderately expanded, narrowest in mid-shaft [20]. The ulnar and radial condyles are positioned anteriorly as in most therizinosaurians [2, 8]. Close to the lateral surface of distal ends, there is a significant posterior tuberosity opposite the radial condyle. Next to the tuberosity and extending laterally is a dorsoventrally elongate ectepicondyle. The entepicondyle is hypertrophied and extends anterolaterally. On the posterior surface of the distal end, there is a ridge originating from the hypertrophied

entepicondyle. Concordant with this ridge, there is a groove on the posterior surface closer to the medial side, which separates the condyles of the distal end.

Ulna

Both ulnae are partial and preserve the distal aspect. The left ulna is more complete including the posterior surface (Figs 6B, 7D). Although a proximal end of the right ulna was noted as being present by Xu, et al. [8], the element could not be found at the IVPP during this study. Based on the preserved portions, the ulna is estimated to be shorter than the humerus, as in most therizinosaurians. The ratio of the ulna to the humerus in early-branching therizinosaurians such as *Falcarius* and *Jianchangosaurus* is 77~78% [2, 20], and *B. inexpectus* might be close to this ratio. Among late-branching therizinosaurians the ratio of these elements is 72% in *Nothronychus* [26] to as elongate as 85% in *Erliansaurus* [32]. The preserved parts and the impression show that the mid-shaft of the ulna is straight and not bowed, like in most other therizinosaurians such as *Jianchangosaurus*, *Nothronychus*, *Erliansaurus* and *Therizinosaurus* [20, 26, 32], but different from that of *Falcarius* which is bowed [2]. The cross-section of the ulnar shaft of *Beipiaosaurus* is oval medially, becoming flattened near the distal end and is semicircular in distal view. There is prominent, medially projecting tubercle on the distal end, which is anteroposteriorly flattened, similar to *Falcarius* [2].

Radius

Both distal radii are preserved, along with the proximal end of the right radius. (Figs 6B, 6C, 7D). Although the diameter of the radius is larger than ulna, it is still gracile in shape. A gracile radius is also present in *Falcarius*, *Jianchangosaurus*, and *Neimongosaurus* [2, 20, 23]. The radius of later-branching therizinosaurians are more

massive and robust [4, 5]. Based on the preserved parts and the impression, the radius of *B. inexpectus* is a relatively straight element, like *Falcarius* and *Jianchangosaurus* [2, 20]; whereas in *Therizinosaurus* and *Neimongosaurus*, this element is relatively sigmoidal [2, 4, 23]. The proximal end of the radius is oval and medially deflected to form the proximal fossa. The cross-section of the shaft is subcircular, becoming subtriangular and mediolaterally expanded approaching the distal end, which flattens medially to articulate with the distal ulna.

Carpus

Nine elements of the carpus are preserved. Present are distal carpals II, III, and IV, the intermedium, and the radiale of the left wrist, and distal carpal III and IV, the intermedium, and the radiale of the right wrist (Figs 6D, 7C and 7E).

The radiale of the left manus is V-shaped, with a depression on the proximal end, near the contact with the radius (Fig. 7E; [8]). Positionally, the right radiale is also preserved near the distal carpal III with a rod-like shape (Fig. 6D), but the poor preservation hinders the further identification and description. Both intermedia are preserved. Considering both of the elements, the intermedium of *B. inexpectus* is a flat and round element with a wedge-shaped, narrower end. Intermedia are either fragmented or not exposed well, so the articulation with the other carpals, and the precise morphology, are difficult to identify.

There are morphological and positional discrepancies in the preserved carpals that prevent us from confident identification of these elements. We present both hypotheses here.

If we identify the distal carpals by their preserved position relative to the metacarpals. The distal carpal II of *B. inexpectus* is large and oval [8], and it is unfused

to distal carpal II. Whether this is related to ontogenetic stage is unclear. In *Falcarius*, distal carpals II and III are partially fused dorsally, and together these elements make up the semilunate [2]. The distal carpals of *B. inexpectus* differ from those of *Alxasaurus*, in which the distal carpal II is the largest [6]. In *B. inexpectus*, distal carpal III is larger than distal carpal II. distal carpal III is slightly elongate and caps primarily the proximal surface of metacarpal III and touches the proximal surface of metacarpal II [8]. In proximal view, distal carpal III is convex dorsally and ventrally, which composed a well-defined trochlear groove for the radiale. Although the semilunate is smaller and the anterior process is blunter and more reduced, the overall shape of it is identical to that of *Deinonychus* [8, 36]. In early-branching therizinosaurians such as *Falcarius*, the fused distal carpal II and III is larger and more robust than other therizinosaurians and dromaeosaurs [2].

However, we recognize a different possibility for the identifications of distal carpal II and III. Morphologically, it seems that the carpal element termed the “semilunate” in Xu et al. [8] and also labelled as “dc III” on our intext figures is most similar to the single carpal distal carpal II in *Falcarius*, and that the “semilunate” carpal structure would be comprised of both distal carpal II and distal carpal III in early branching therizinosaurs [2, 37]. Also, based on the size ratio of the distal carpals and the morphology of “semilunate” in *B. inexpectus*, this element is more identical to that of the distal carpal II of *Alxasaurus* [6, 37]. In this case, it is highly possible that the positionally consistent distal carpal II and III are translocated during the preservation or the preparation, and they are actually distal carpals III and II, respectively.

The distal carpals IV are also incomplete on both manus. The distal carpus IV is a proximodistally thin element, and tapers on the lateral end giving it a subtriangular shape. The articular facet of the distal IV is slightly concave. The preservation of the

distal carpal IV of both hands are incomplete, and it is missing in other therizinosaurian taxa, so there is little else to support the identification of this element.

Manus

The manus of IVPP V11559 is relatively complete, and missing parts can be inferred from impressions (Figs 6D, 7A). In general, the manus of *B. inexpectus* is elongate and slender, with well-developed ginglymoid articulations and raptorial unguals. Digit IV is shorter than digit III.

Metacarpus

All the metacarpals of *B. inexpectus* are preserved, except for the left metacarpal IV, (Figs 6D, 7A). In general, metacarpal elements of *B. inexpectus* is resemble to those of *Falcarius* [2].

Metacarpal II is the most robust and shortest of the metacarpals, only half the length of metacarpal III. The cross-section is subtriangular with a laterally flattened facet for articulation with metacarpal III. There is a concavity on the proximal surface of metacarpal II that extends to the dorsal surface between the medial process and the dorsal process, forming a notch. There are three processes on the proximal articular surface, which make a triradiate shape. This condition is also present in the early-branching therizinosaurian *Falcarius* [2]. Among the three processes, the medial process extends farthest to support the dorsomedial extension of distal carpal I. The same condition can also be seen in *Falcarius* and *Jianchangosaurus* [2, 20], but not as extremely as in *Erliansaurus* [32]. The lateroventral surface of the proximal end of metacarpal II is pointed and extends to underlie the base of metacarpal III; this is known as the "rectangular buttress". This character was first described in *Alxasaurus* and as a

synapomorphy for Therizinosauroidea [6]. Although this buttress is also present in the earlier-branching *Falcarius*, it is more moderate [2]. The distal end of metacarpal II is slightly broader and asymmetrical, with a more extensive lateral condyle and a medially rotated axis. This feature is also seen in *Falcarius*, *Jichangosaurus*, and *Erliansarurus* [2, 20, 32]. As in *Falcarius* and *Therizinosaurus,* the distal end of metacarpal II lacks collateral ligament pits and a dorsal extensor pit.

Metacarpal III is the longest of the metacarpals, and has transversely expanded and robust ends. The shaft is rectangular proximally and becomes oval and dorsoventrally flattened in the midshaft. The proximal articular surface is rectangular and convex, unlike the proximal articulation surface of metacarpal II, which is concave. Each margin of the rectangular proximal surface is divided by rounded tuberosities, and a dorsolateral tuberosity extends to overhang the proximal end of metacarpal IV. This morphology is similar to that of *Falcarius* [2]. The distal end of metacarpal III is asymmetrical, with a vertically oriented, rounded lateral condyle and a larger medial condyle that is angled outward. A larger medial condyle is also described in *Falcarius* and *Erliansaurus*, but in *Falcarius* it is less prominent [2, 32].

Metacarpal IV is only preserved on the right manus. By observing the impression, metacarpal IV can be inferred to be a thin, long, and straight element, more gracile than metacarpal II and metacarpal III. This character is similar to that of *Faclarius* morphologically [2]. The length of MCIV is about 75% of Metacarpal III. The shaft of metacarpal IV is oval in cross-section. The proximal end of metacarpal IV is convex and subtriangular; whereas the distal end is slightly bowed toward metacarpal III.

Manual phalanges

The left arm of *B. inexpectus* is more complete than the right one. There are many

missing and incomplete right phalanges, but clear impressions of these missing elements remain (Figs 6D, 7A). There are well-developed ligament pits on the lateral sides of distal ends. All the manual unguals are deep, compressed mediolaterally, and curved strongly [6, 8]. As in *Falcarius*, the collateral grooves of the manual unguals are not continuous with large depressions in the medial and lateral surface of the proximal aspect [2]. In *Lingyuanosaurus* and *Jianchangosaurus* these features connect [21]. Collateral grooves rise dorsally toward the distalmost tip, as in many other therizinosauroids, including *Lingyuanosaurus*, *Alxasaurus*, and *Therizinosaurus* [6, 21]. Manual unguals are subequal in length with the penultimate phalanges in *B. inexpectus*. Unlike, in the therizinosaurids *Nothronychus* and *Therizinosaurus* in which the unguals are longer than the penultimate phalanges ([2], fig. 5; [31], fig. 1).

Digit II is the shortest of the three digits, but phalanx II-1 is the longest and the most robust phalanx. The same condition can also be seen in *Jichangosaurus* and *Falcarius* [2, 20]. The proximal articular surface of phalanx II-1 is subtriangular and taller than wide. Although the midshaft is missing, from the impression and the preserved distal shaft, it can be inferred that the midshaft is straight with a subcircular cross-section. The distal ginglymoid articular surface is symmetrical with a deep intercondylar groove, and the condyles are more extensive ventrally than dorsally. Collateral ligament pits are well developed and positioned more dorsally. The manual ungual of digit II of most therizinosaurians is the longest and largest including *Jianchangosaurus* [20], *Alxasaurus* [6], and *Erliansaurus* [32]. However, the manual ungual II of *B. inexpectus* is slightly shorter than manual ungual III, and it possesses the strongest degree of curvature. As in other early-branching therizinosaurians, *B. inexpectus* also possesses a deeper proximal articular surface and more massive flexor tubercle similar to that of oviraptorosaurians [2, 6, 32]. The flexor tubercle and the

ventral margin of the proximal articular surface of PII-2 are separated by a narrow and deep groove.

Digit III is the longest digit, PIII-1 is about 80% length of phalanx II-1, and phalanx III-2 is only slightly shorter than phalanx II-1. phalanx III-1 is stout in shape, with a hexagonal proximal surface. In proximal view, the dorsal intercondylar tuberosity is less pronounced, but the ventral intercondylar tuberosity is relatively robust. On the distal end, collateral ligament pits are poorly developed and the pits position nearly centered on the condyle. In phalanx III-2, the cross-section of the proximal end is oval, and dorsoventrally taller than wide. The shaft of phalanx III-2 of *B. inexpectus* is straight, and gradually tapers in dorsoventral height toward the distal end. Nonetheless, it remains taller than wide in cross-section. On the distal end of phalanx III-2, the intercondylar groove is deep, the condyles are taller than the height of the mid-shaft, and the collateral ligament pits are located more dorsally. These features of distal end are characteristic of penultimate phalanges [2]. Manual ungual III is less re-curved than manual ungual II, but longer. A longer second claw is a character similar to that of *Archaeopteryx* and *Protarchaeopteryx* [8, 38]. The proximal articular surface and flexor tubercle of PI-3 are divided by a broad and shallow notch. The flexor tubercle of manual ungual III is narrower and smoother than that of manual ungual II, and lacks the distal pits present in manual ungual II. On the tip of the left ungual III, there is a dorsoventrally flat extension which might represent preservation of the keratin sheath. If so, the claw sheath of *B. inexpectus* seems to be more flattened, rather than a sharply pointed structure, but this might be caused by taphonomy process.

Digit IV is relatively gracile, with extremely shortened phalanges IV-1 and phalanx IV-2. When combined, the length of these two phalanges is equal to that of phalanx IV-3 [8]. Digit IV is in poor condition, most parts of phalanx IV-1 and phalanx

IV-2 are missing, so it is difficult to observe any detailed structure. phalanx IV-3 is relatively complete. The shape of phalanx IV-3 is gracile and elongate, with a mediolaterally narrow and oval cross-section. The collateral ligament pits of the distal end are well developed and located dorsally. phalanx IV-4 is the smallest manual ungual of the three digits. The flexor tubercle, the groove that separates it from proximal joint surface, as well as the lateral sulci of phalanx IV-4, are the most poorly developed of any manual ungual.

Pelvic Girdle

Pelvic girdle of IVPP V11559 preserved partial ilium, pubis and ischium in materials from reported in 1999 [8], and materials found by the re-excavated of the same quarry and reported in 2003 remained a complete ilium and two incomplete ischia (Fig. 8, Table 3) [11]. In the pelvic girdle of *B. inexpectus* processes features such as parallelogram ilium and propubic, which are distinct more primitive than late-branching therizinosaurids [3, 8].

Figure 8. Photographs of pelvic girdle elements of *B. inexpectus* (IVPP V 11559) A. left ilium in medial view with pubis; B. partial ilium and ischium; C. right ilium in lateral view; D. right ischium in lateral view and left ischium in medial view. **Abbreviations: ac**, acetabulum; **ant**, antitrochanter; **I.isp**, ischiadic peduncle of ilium; **I.pp**, pubic peduncle of ilium; **Is.ip**, iliac peduncle of ischium; **l.Il**, left Ilium; **l.Is**-left ischium; **op**, obturator process; **poa**, postacetabular; **pra**, preacetabular; **pran**, preacetabular notch; **Pu**, pubis; **r.Is**, right ischium; **ve**, ventral extension. Scale bars equal 2 cm.

Table 3. Measurements in millimeters of pelvic girdle and hindlimb elements of *B. inexpectus* (IVPP V 11559)

Element	Total length	Hight	Note

Ilium, right	>115.8	80.1※	※From acetabulum
Pubis	>233.3※		※losing distalmost boot part
Ischium	>150.0※		※losing proximal end
	**Length**	**Width, proximal**	**Width, distal**
Femur, right	275.8	92.1	56.1
Tibia, right	271.2	-	65.7
	**Length**	**Width**	
Astragalus	-	56.7	
Calcaneum	32.5	20.3	
MTI	31.4	12.6	
MTII	96.5	25.3	
MTIII	105.7	26.3	
?PI-1	36.3		
Pedal ungual	51.7		

Ilium

The left ilium of *B. inexpectus* is partially preserved and exposed in medial view (Figs 8A-B). The right ilium was found in the re-excavation. It is relatively complete and exposed in lateral view (Fig. 7C). The ilium is parallelogram in shape, similar to that of dromaeosaurids and early-branching birds, but different from that of late-branching therizinosaurians which have an ilium more similar in shape to sauropods [8, 39]. The length of the pre- and postacetabular portions of the ilium are subequal, as in many oviraptorosaurians and early-branching ornithomimosaurians [3, 8, 40, 41]. The relative proportion of the pre- and postacetabular aspects of the ilium is difficult to quantify in other therizinosaurians except for some taxa, such as *Falcarius*, for which the preacetabular portion is approximately 30% longer than the postacetabular portion. *Nothronychus* and *Segnosaurus* also possess longer preacetabular portions, but the ratio

is not as extreme as in *Falcarius* [19, 31].

The preacetabular blades of therizinosaurians are dorsoventrally deep, dubbed the "altiliac" condition, and bear a pointed ventral extension. This extension is extreme in *Lingyuanosaurus*, *Segnosaurus*, and *Nanshiungosaurus*, extending ventral to the dorsal margin of acetabulum [3, 21, 42]. However, this character is less significant in *B. inexpectus*, in which case the ventral aspect of the preacetabular portion of the ilium only extends slightly ventral to the dorsal acetabulum [11]. Early-branching therizinosaurians such as *Falcarius* and *Jianchangosaurus* lack this feature [19, 20]. The preacetabular notch is considerably higher than the acetabulum as in other therizinosaurians including the early-branching *Falcarius*, but in theropods, the dorsal margin of acetabulum is subhorizontal with the ventral aspect of the preacetabulum [19].

The supracetabular crest is relatively mediolaterally deep, and entirely capped the femoral head. The dorsoventral height of the postacetabular portion gradually reduces posteriorly in lateral view. It is subrectangular in shape, with a blunt posteroventral corner. This feature is already present in the earliest-branching therizinosaur *Falcarius*, and is a character of all therizinosaurians. The posteroventral aspect of the ilium is deflected laterally at a right angle to the vertical ramus. The brevis fossa is shallow, subcircular and oriented mediolaterally, which is similar to coelurosaurians generally [8, 43].

The pubic peduncle of *B. inexpectus* is longer than the ischiadic peduncle, as in therizinosaurians, dromaeosaurids and *Archaeopteryx* [8, 43], but the length is subequal in earlier-branching therizinosaurians (e.g., *Falcarius*; [19]). The pubic peduncle is equidimensional, unlike that of earlier-branching *Falcarius* and *Jianchangosaurus*, in which the anteroposterior length is as twice that of the the mediolateral width; whereas in later-branching therizinosaurians, the mediolateral width is two or three times greater

than the anteroposterior length ([19]; [20], fig 9). *Beipiaosaurus inexpectus* is intermediate between these values. The middle of the pubic peduncle is posteriorly concave, which is also seen in other late-branching therizinosauroids, including *Lingyuanosaurus*, *Suzhousaurus*, and *Nanshiungosaurus* [21, 22, 30]. The articular facet of pubic peduncle is oriented anteroventrally, which when combined with the morphology of the proximal pubis creates a propubic pelvis. The ischiadic peduncle is peg-like. These characters are similar to *Falcarius* and *Jianchangosaurus* [19, 20]. There is a prominent antitrochanter on the dorsolateral surface of the acetabulum, which rounds out the acetabulum in lateral view.

Pubis

Both pubes are preserved, lacking proximal and distal ends (Fig. 8A). There is a slight curvature of the distal shaft creating a sigmoid shape. The shaft is anteroposteriorly compressed and drop-shaped in cross-section as in *Falcarius* [19], but different from that of other late-branching therizinosaurians including *Alxasaurus*, *Segnosaurus*, *Nothronychus*, and *Shuzhousaurus* which have more transversely flattened shafts [8, 30, 31]. The pubic apron is compressed anteroposteriorly, and might have extended more than half the length of the shaft as in *Falcarius* [19], but due to the fact that both ends are missing, the proportions cannot be confidently calculated. The pubic boot in *B. inexpectus* is as of yet unknown. The ischial boot from the 2003 specimen was erroneously compared to the pubic boot of *Falcarius* in Zanno [19]; this error was subsequently corrected in Zanno [3].

Ischium

Two incomplete ischia are present, preserving the proximal portion of the right

ischium and the shaft and distal portions of both the right and left ischia ([11], Fig. 8D; [8], Fig. 8B). The ischium is estimated to be subequal or slightly shorter than the pubis. The relative length of the ischium is shortest in *Falcarius*, which is only about 2/3 length of pubis; in *Jianchangosaurus*, the ischium is about 20% shorter; and in late-branching therizinosaurians, the length of these two elements are subequal [19, 20].

Ischia are slightly curved posteriorly and mediolaterally compressed. The lateral surface is slightly convex and the medial surface is flattened. The proximal portion is damaged, but bears a fan-shaped expansion in lateral view, and might be similar to *Faclarius*, which possesses a longer iliac peduncle than pubic peduncle [19].

On the anterior surface of the mid-shaft, closer to the proximal end, there is a prominent, dorsoventrally expanded obturator process. It is different from the condition of late-branching therizinosaurians, which have a hypertrophied and distally positioned obturator process [19, 20]. The distal point of the obturator process in *B. inexpectus* first projects anterodorsally and then anteroventrally, forming a sinusoidal process, unlike in *Falcarius* and *Jianchangosaurus*, in which it points anteriorly [19, 20]. The position of obturator process in *B. inexpectus* and *Jianchangosaurus* is relatively close to the mid-shaft, which is similar to that of tyrannosaurs and early-branching ornithomimosaurs [41, 44]. On *Falcarius*, the obturator process is positioned more distally, as in dromaeosaurs, troodontids, early-branching birds, and oviraptorosaurs [18, 19, 36, 40, 45-50].

The distal end of ischium bears a prominen, anteriorly expanded, and semicircular boot, in medial view. The anterior margin is relatively pointed and convex, and the posterior margin is round and blunt. A prominent distal ischial boot is different than that observed on *Jianchangosarus*, *Alxasaurus*, and *Nothronychus*, in which the boot is only expanded or thickened slightly [6, 20, 27].

Hindlimb

Only the right leg is preserved on IVPP V11559; it is relatively complete (Fig. 9, Table 3). Although Xu, Tang, and Wang [8] describe the tibia of IVPP V 11559 as being longer than the femur, our measurement shows that they are subequal in length and the femur is slightly longer (Table 3). The hindlimb of *B. inexpectus* is intermediate in morphology, including the relative ratios of the hind limb elements. The ratio of femur/tibia and metatarsus/tibia in therizinosaurians is as follows: *Falcarius* 91/45%, *Jianchangosaurus* 65/54%, *B. inexpectus* 101/39%, *Nothronychus graffmi* 104/35%, *Neimongosaurus* 118/37%, *Erliansaurus* 110%/unknown, and *Segnosaurus* 102/32% [8, 19, 20]. The ratio in *B. inexpectus* fits its phylogenetic position between earliest-branching taxa (*Falcarius* and *Jianchangosaurus*) and therizinosaurids.

Figure 9. Photographs of hindlimb elements of *B. inexpectus* (IVPP V 11559) A. right femur in anterior view and tibia in posterior view; B. left tibia and fibula; C. right tarsus and pedal elements. **Abbreviations:** ap, ascending process; **As**, astragalus; **asc**, astragalus condyle; **bo**, body; **Cal**, Calcaneum; **Dt**, distal tarsal; **F. lc**, lateral condyle of femur; **F. mc**, medial condyle of femur; **fc**, fibular crest; **fh**, femoral head; **Fi**, fibula; **lm**, lateral malleolus; **lt**, lesser trochanter; **mct**, medial crest; **mm**, medial malleolus; **MT I-III**, metatarsus I-III; **?PI-1 to ?PIV-3**, ?phanlanges I-1 to IV-3; **T.mc**, medial condyle of tibia. Scale bars equal 2 cm.

Femur

The right femur is preserved and exposed in anterior view (Fig. 9A). Both *B. inexpectus* and *Falcarius* possess relatively gracile femora that display a mosaic of late-branching maniraptoran and early-branching coelurosaurian features [19]. The femur of *B. inexpectus* is somewhat intermediate between the recurved condition of *Falcarius*

and the early-branching straight femur of many other therizinosauroids, including *Jianchangosaurus*, *Lingyuanosaurus*, *Erliansaurus* and *Neimongosaurus* [20, 21, 23, 32].

The femoral head of *B. inexpectus* in anterior view is perpendicular to the long axis of the shaft, similar to *Falcarius* and *Jianchangosaurus* [19, 20]. This feature is different from that of late-branching therizinosaurians, such as *Lingyuanosaurus*, *Alxasaurus* and *Nothronychus*, which possess a dorsomedial rotated femoral head [6, 21, 31]. The femoral head is large and bears a distinctly rounded head and weakly constricted neck, similar to the condition in *Jianchangosaurus*, but different than *Falcarius* and *Lingyuanosaurus*, which have *a* more distinct and constricted femoral neck [19, 21]. As in *Falcarius* and *Jianchgosaurus*, the femoral head of *Beipaiosaurus* is continuous with the greater trochanter. This is unlike the condition in late-branching therizinosaurians such as *Lingyuanosaurus*, *Nothronychus* and *Neimongosaurus*, which have a depression between the femoral head and the greater trochanter in anterior view [19-21]. This depression is not only present in late-branching therizinosaurians, but also in oviraptorosaurians and alvarezsaurs [19, 40, 51]. However, the absence of this feature in early-branching therizinosaurians indicates that it is not synapomorphic of these clades.

The lesser trochanter of *B. inexpectus* is wing-like and separated from the greater trochanter by a narrow and deep cleft, which is a character present in early-branching coelurosaurs [8, 19]. Both *Falcarius* and *B. inexpectus* possess an alariform lesser trochanter, but the shape is cylindrical in *Alxasaurus* and *Jianchangosaurus*, and even reduced in *Alxasaurus* [6, 19, 20]. The fourth trochanter projects from the posterior surface of the femur, with a ridge shape which is transversely thin and proximodistally long. The cross-section of the shaft of femur is subtriangular.

In anterior view there is a slight depression separating the medial condyle and the lateral condyle on the distal femur. In anterior and distal view, the distal condyles are asymmetrical, with a slightly larger and more extensive medial condyle than lateral condyle, which is opposite to that of *Falcarius*, which possesses a more extensive lateral condyle [19].

Tibia

Both tibiae are preserved, and the right tibia is relatively complete (Figs 9A-B). In general, the shape of the tibia of *B. inexpectus* is gracile and slender, with some proportions which are more primitive than those of late-branching therizinosaurians.

The posterior surface of the right tibia of *B. inexpectus* is exposed and bears an asymmetrical proximal end in posterior view. On the proximal end, the medial condyle is dorsally higher and positioned more proximally, which is similar to that of *Falcarius* [19]. This asymmetrical proximal end in *Falcarius* corresponds with the less extensive medial condyle of femur. However, this is not the case for *B. inexpectus* because the medial condyle of the femur is more distally extensive.

The tibia has a relatively straight shaft and its cross-section changes from subtrangular proximally to anteroposteriorly flattened distally. Due to the preservation position, the lateral side of the right tibia cannot be observed, but in the partial left tibia, there is a prominent fibular crest extending to the mid-shaft. Although it is damaged, it can be determined that it is more developed than *Falcarius* [19], and less developed than in late-branching therizinosaurians such as *Nothronychus* and *Erliansaurus*, which have fibular crests that extend distally to the mid-shaft [31, 32].

The distal articular facet of tibia of *B. inexpectus* is anteroposteriorly narrow and transversely broad, with a blunt and robust medial malleolus, similar to *Falcarius*. But

the medial malleolus of *Falcarius* is orientated anteromedially [19], whereas in *B. inexpectus* it is orientated medially. The lateral malleolus is large and semicircular in shape to support the fibula.

Fibula

An incomplete left fibula is preserved, lacking the distal end (Fig. 9B). In general, the fibula is much slenderer and more gracile compared to tibia, especially the distal portion. The fibula of *B. inexpectus* lacks a medial fossa on the proximal portion, as in other therizinosaurians, oviraptorosaurians, and dromaeosaurs [19, 26, 52]. Similar to *Alxasaurus* and some avialans, the medial surface of fibula is relatively flattened [6, 8, 53].

Tarsus

The right astragalus, calcaneum and a distal tarsal are preserved and exposed in posterior view (Fig. 9C). They are unfused with the distal tibia, proximal metatarsals, and with each other. Lack of fusion may be the result of ontogeny, but in *Faclarius* these elements are also not fused even in the largest individual [19]. As in other therizinosaurians, the astragalar condyle is reduced, so it only capped a portion of the distal end of the tibia. In *Faclarius*, the astragalus and the calcaneum are relatively larger and cap the distal tibia and fibula entirely [5, 8, 19, 31].

The astragalus consists of an asymmetrical and robust, saddle-shape body, with a significantly larger medial surface. In posterior view, the medial aspect is thick proximodistally, and gradually narrows laterally. This condition is shared between *B. inexpectus* and *Falcarius* [19]. However, reduction of the astragalar body in late-branching therizinosaurians causes this feature to be absent in late-branching members

[19]. As in other therizinosaurians, *B. inexpectus* also possesses a tall ascending process. The ascending process and the medial margin of the body is inset, with the base of ascending process rising vertically.

The calcaneum is sub-oval and disk-shaped [8], with a slightly convex surface on the medial side. According to Xu et al. ([8], fig 2e), a single distal tarsal is present, which attaches to the proximal surface of metatarsal IV. However, this element can no longer be located.

Metatarsus

Five elements of the right metatarsus are preserved, and in relatively complete condition (Fig. 9C) [8]. However, due to the position of preservation, only portions of these elements are exposed and can be observed. As in *Falcarius* and *Jianchangosaurus, B. inexpectus* has a typical functionally tridactyl, and relatively gracile pes, in contrast to late-branching therizinosaurians which have a stout and tetradactyl pes [8, 19, 20]. The metatarsus of *B. inexpectus* is compact proximally and elongate in general (~39% the length of the tibia). This ratio is longer than other late-branching therizinosaurians but shorter than *Falcarius* and *Jianchangosaurus,* as well as most theropods in which this ratio is typically over 45% [6, 8, 19, 20].

Metatarsal I of *B. inexpectus* is relatively robust, with a tapered and flattened proximal portion for articulation with metatarsal II. Metatarsal I articulates with metatarsal II on the mid-shaft and does not contact the tarsus, in contrast to the functionally tetradactyl pes of late-branching therizinosaurians.

In medial view, metatarsal II is slightly sigmoidal, with a proximal portion that is slightly curved anteriorly and a distal portion that curves posteriorly. In proximal view, the proximal articulation surface is mediolaterally narrow, oval in shape, and the lateral

surface is flattened to correspond to the medial surface of metatarsal III. Metatarsal II is slightly narrower anteroposteriorly in the mid-shaft, especially the anterior surface, which slopes more than the posterior side, but it is still in the shape of oval which anteroposteriorly length longer than width in general and expanded distally. The distal end of Metatarsal II of *B. inexpectus* is fan-shape in medial view and expanded distally, with a smooth medial surface, unlike *Falcarius*, which has a subcircular distal end and collateral ligament pits [19].

Metatarsal III and metatarsal IV are longer than metatarsal II. metatarsal III is the slenderest and the longest of the metatarsals, and it is transversely compressed. According to Xu et al. ([8], fig 2e), the metatarsal IV and metatarsal V are preserved. However, metatarsal V and the proximal end of metatarsal IV can no longer be located.

Pedal phalanges

Pedal elements are disarticulated and partially broken, so it is difficult to identify them. Five pedal phalanges are preserved and exposed relatively well, and we tentatively identify them as phalanxes I-1, I-2, II-1, III-1, and IV-2 or 3 (Fig. 9C).

A well-exposed phalanx near metatarsal I is tentatively identified as phalanx I-1. This phalanx is relatively robust, with a subcircular proximal articulation surface. The cross-section becomes narrower from the proximal to the distal mid-shaft. Both the ginglymoid articular surface and extensor ligament pits are poorly developed on the distal end, but the collateral ligament pits on the sides are deep and well-developed and positioned centrally. The distal articular surface of this phalanx is relatively symmetrical. A single pedal ungual is preserved next to the referred phalanx I-1 which we interpret as phalanx I-2. It is short and curved, and smaller than any manual ungual [8].

Near the referred phalanx I-1, there is an elongate element interpreted as phalanx II-1, which possesses a subcircular proximal articular surface. Although it is poorly developed, an extensor ligament pit can be seen on the ventral side of the distal end. The shaft is curved laterally in ventral view. Articulating with the distal end of this phalanx, there is a shorter, more robust phalanx, likely to be phalanx II-2, and beyond this the proximal portion of phalanx II-3. Beneath the second digit rests another phalanx that we tentatively identify as phalanx III-1. This distal portion of this phalanx is exposed in lateral view and the collateral ligament pits of it are shallower than phalanx I-1.

Discussion

It has been more than twenty years since *B. inexpectus* was first described. Since this time, multiple new early-branching and late-branching therizinosaurs have been discovered and described (e.g. *Falcarius* [7], *Jianchangosaurus* [20], *Lingyuanosaurus* [21], *Nothronychus* [26]). In addition, new materials of *B. inexpectus* and *Beipiaosaurus sp*. have been published, including the recovery and description of additional materials of the holotype representing the pelvic girdle elements, sacrals, and a series of nearly complete caudals (Xu et al. [11]. A new feather type was additionally described from around the tail of the holotype of *B. inexpectus* and the neck of a new specimen, *Beipiaosaurus*. *sp.* (Xu et al. [12]. Based on these new discoveries, Zanno [19] revised the autapomorphies of *B. inexpectus*. This diagnosis was further revised after a cranial osteology was published by Liao and Xu [13] and several cranial diagnostic features were added. Although new taxa of therizinosauroids have since been described, the autapomorphies proposed by Zanno [19] and Liao and Xu [13]

appear to remain valid.

New acquired diagnostic features

In this study, we propos three new autapomorphies for *B. inexpectus*.

Ungual II shorter than III: When measuring ungual length as a straight line from the tip of the ungual to the ventralmost portion of the articular facet, the ungual of digit three is the longest one in *Beipiaosaurus inexpectus*. This feature differs from other therizinosaurians, including *Jianchangosaurus*, as well as later-branching *Alxasaurus* and *Erliansaurus* [2, 6, 20, 32], in which the ungual of digit two is the longest.

The pre- and postacetabular length are subequal: In *Beipiaosaurus inexpectus*, the pre- and postacetabular of ilium are subequal in anteroposterior length. This feature is similar in oviraptorids and early-branching ornithomimids [8, 19, 40, 41], but differs from other therizinosaurians, which possess relatively longer preacetabular portions of the ilium, including the earliest-branching member *Falcarius* and later-branching *Nothronychus* and *Segnosaurus* [19, 31].

Equidimensional pubic peduncle of ilium: In *Beipiaosaurus inexpectus*, the anteroposterior length and width of the public peduncle is approximately the same. This is a transitional feature linking early-branching to late-branching members, and although it is found in other coelurosaurs, it is currently unique among therizinosaurs. In earlier-branching therizinosaurians, such as in *Falcarius* and *Jianchangosaurus* ([19]; [20], fig. 9), the anteroposterior length is greater than its width, whereas in later-branching members (e.g. *Suzhousaurus* [30], *Nanshiungosaurus* [22], and *Segnosaurus* [54]), the width is significantly greater than its length.

New proposed synapomorphies for therizinosaurians

Here we discuss three possible synapomorphies found in this study—two for Therizinosauroidea and another for Therizinosauridae.

Straight ulna: In *B. inexpectus*, the ulna is straight as in most other therizinosaurian taxa (including *Jianchangosaurus*, *Alxasaurus*, *Nothronychus*, *Erliansaurus*, and *Therizinosaurus* [6, 20, 26, 32], but the ulna of the earliest-branching member *Falcarius* is bowed [2]. Therefore, a straight ulna appears to be a synapomorphy of Therizinosauroidea, and a reversal among coelurosaurs.

Among other coelurosaurian dinosaurs, a straight ulna is present in medium-bodied (e.g., *Sinornithomimus* [55]) and large-bodied taxa (e.g., some ornithomimids and *Gallimimus* [56], *Tyrannosaurus* [44], and *Gorgosaurus* [57]). In many smaller sized maniraptoran dinosaurs (e.g., *Microraptor* [47], *Heyuannia* [58], and *Confuciusornis* [59]), the ulna is bowed as in *Falcarius*.

Blunt termini of furcula: In *B. inexpectus*, *Falcarius* [19], and *Jianchangosaurus* [20], the furcular termini taper to a point. This is different from the robust and blunt termini in *Neimongosaurus* [23]. The blunt and robust condition may be a synapomorphy of Therizinosauridae. It is likely that the evolution of blunt termini has functional implications; however, further research is needed to understand the impact of this trait. Moreover, it is difficult to determine whether this feature is a synapomorphy for Therizinosauridae or just an autapomorphy for *Neimongosaurus* because currently the preservation of this element is rare among the clade.

Broad intraclavicular angle: As mentioned by Zanno [3], *Falcarius* possesses an acute angle (approximate 104 degrees) compared to *B. inexpectus* and *Neimongosaurus* (approximately 150 degree). Therefore, a wider intraclavicular angle may be a synapomorphy for Therizinosauroidea.

Conclusions

Since first reported in 1999 and 2003, the holotype of *B. inexpectus* has only been briefly described. In our restudy of this specimen, we not only provide a detailed description and illustrations of the known elements, but describe materials of the holotype that are not mentioned in previous papers (e.g., antero-middle dorsal and middle dorsal. Our morphological observations support previous proposals for the phylogenetic position of *B. inexpectus* as the earliest-branching member of Therizinosauroidea.

We also expand the diagnosis of this taxon by adding three additional autapomorphies of the postcranial skeleton (ungual II shorter than III, subequal length of the pre- and postacetabular, and equidimensional pubic peduncle of ilium). Furthermore, we describe new synapomorphies for more inclusive therizinosaur taxa.

Detailed information on the osteology of the postcranial skeleton of *B. inexpectus* is important for understanding the evolution of therizinosaurs since the majority of therizinosaur taxa are known exclusively from postcranial materials. The updated diagnosis and possible synapomorphies proposed in this study shed new light on the phylogeny and alpha taxonomy of Therizinosauria.

Acknowledgements

The authors would like to thank Ding Xiaoqing for preparing the specimen and Qin Zichuan (Bristol University) for valuable comments on the early versions of the manuscript.

Reference

1. Clark JM, Perle A, and Norell MA. The skull of *Erlicosaurus andrewsi,* a Late Cretaceous "segnosaur" (Theropoda: Therizinosauridae) from Mongolia. American Museum Novitates. 1994; 3115:1-39.
2. Zanno LE. The pectoral girdle and forelimb of the primitive therizinosauroid *Falcarius Utahensis* (Theropoda, Maniraptora): analyzing evolutionary trends within Therizinosauroidea. Journal of Vertebrate Paleontology. 2006; 26: 636-50.
3. Zanno LE. A taxonomic and phylogenetic re-evaluation of Therizinosauria (Dinosauria: Maniraptora). Journal of Systematic Paleontology. 2010; 8: 503-43.
4. Barsbold, R. New data on Therizinosaurus (Therizinosauridae, Theropoda). Transactions, Joint Soviet-Mongolian Paleontological Expedition. 1976; 3: 76–92.
5. Perle A. Segnosauridae - A new family of theropods from the Late Cretaceous of Mongolia. Transactions, Joint Soviet-Mongolian Paleontological Expeditions, 1979; 8: 45-55.
6. Russell DA, Dong ZM. The affinities of a new theropod from the Alxa Desert, Inner Mongolia, People's Republic of China. Can J Earth Sci. 1993; 30: 2107-27.
7. Kirkland JI, Zanno LE, Sampson SD, Clark JM, and Deblieux DD. A primitive therizinosauroid dinosaur from the Early Cretaceous of Utah. Nature. 2005; 435: 84-7.
8. Xu X, Tang ZL, and Wang XL. A therizinosauroid dinosaur with integumentary structures from China. Nature. 1999; 399: 350-54.
9. Xu X, Zhao XJ, and Clark JM. A New Therizinosaur from the Lower Jurassic Lower Lufeng Formation of Yunnan, China', Journal of Systematic Paleontology. 2001; 21: 477-83.
10. Barrett PM. The affinities of the enigmatic dinosaur *Eshanosaurus deguchiianus*

from the Early Jurassic of Yunnan Province, People's Republic of China. Palaeontology. 2010; 52: 681-88.

11. Xu, X, Cheng YN, Wang XL, and Chang CH. Pygostyle- like Structure from *Beipiaosaurus* (Theropoda, Therizinosauroidea) from the Lower Cretaceous Yixian Formation of Liaoning, China. Acta Geologica Sinica. 2003; 77: 294-98.
12. Xu X, Zheng XT, and You HL. A new feather type in a nonavian theropod and the early evolution of feathers. PNAS. 2009; 106: 832-34.
13. Liao C-C, and Xu X. Cranial osteology of *Beipiaosaurus inexpectus* (Theropoda: Therizinosauria). Vertebrata PalAsiatica. 2019; 201957: 117-32.
14. Xu X, Clark JM, Mo J-Y, Choiniere J, Forster CA, Erickson GM, et al. A Jurassic ceratosaur from China helps clarify avian digit homologies. Nature. 2009; 459: 940-44.
15. Feduccia A. The origin and evolution of birds, 2nd ed. Yale University Press: New Haven; 1999.
16. Swisher CC, Wang XL, Zhou ZH, Wang YQ, Jin F, Zhang GY, et al. Further support for a Cretaceous age for the feathered-dinosaur beds of Liaoning,China:New 40 Ar/39 Ar dating of the Yixian and Tuchengzi Formations. Chinese Science Bulletin. 2002; 47: 135-38.
17. Irmis RB. Axial skeleton ontogeny in the Parasauchia (Archosauria: Pseudosauchia) and its implications for ontogenetic determination in Archosaurs. Journal of Vertebrate Paleontology. 2007; 27(2): 350-61.
18. Xu X, and Norell MA. A new troodontid dinosaur from China with avian-like sleeping posture. Nature. 2004; 431: 838-41.
19. Zanno LE. Osteology of *Falcarius utahensis* (Dinosauria: Theropoda): characterizing the anatomy of basal therizinosaurs.Zoological Journal of the

Linnean Society. 2010; 158: 196-230.

20. Pu HY, Kobayashi Y, Lü JC, Xu L, Wu YH, Chang HL, et al. An unusual basal Therizinosaur dinosaur with an ornithischian dental arrangement from northeastern China. PLoS One. 2013; 8: e63423.

21. Yao X, Liao C-C, Corwin S, and Xu X. A new transitional therizinosaurian theropod from the Early Cretaceous Jehol Biota of China. Scientific Report. 2019; 9: 5026.

22. Dong, Z. M. In *Mesozoic and Cenozoic red beds of south China: selected papers from the Cretaceous-Tertiary Workshop*, *Nanxiong*, *Guangdong* Province. (eds Institute of Vertebrate Paleontology and Paleoanthropology & Nanjing Institute of Paleontology Institute of Vertebrate Paleontology) 342–350 (Science Press, 1997).

23. Zhang XH, Xu X, Zhao XJ, Sereno P, Kuang XW, and Tan L. A long-necked therizinosauroid dinosaur from the Upper Cretaceous iren Dabasu Formation of Nei Mongol, People’s Republic of China. Vertebrata PalAsiatica. 2001; 39: 282-90.

24. Clark JM, Norell MA, and Barsbold R. Two new oviraptorids (Theropoda: Oviraptorosauria), Upper Cretaceous Djadokhta Formation, Ukhaa Tolgod, Mongolia. Journal of Vertebrate Paleontology. 2001; 21: 209-13.

25. Zhou ZH, Wang XL, Zhang FC, and Xu X. Important features of *Caudipteryx*-evidence from two nearly complete new specimens. Vertebrata PalAsiatica. 2000; 38: 241-254

26. Kirkland JI, and Wolfe DG. First definitive therizinosaurid (Dinosauria; Theropoda) from North America. Journal of Vertebrate Paleontology. 2001; 21: 410-14.

27. Hedrick BP, Zanno LE, Wolfe DG, and Dodson P. The Slothful Claw: Osteology

and Taphonomy of *Nothronychus mckinleyi* and *N. graffami* (Dinosauria: Theropoda) and Anatomical Considerations for Derived Therizinosaurids. PLoS ONE. 2015; 10: e0129449.

28. Wilson JA. A nomenclature for vertebral laminae in sauropods and other saurischian dinosaurs. Journal of Vertebrate Paleontology. 1999; 19(4):639-653.

29. Senter P, Kirkland JI, and Deblieux DD. *Martharaptor greenriverensis*, a New Theropod Dinosaur from the Lower Cretaceous of Utah. PLoS ONE. 2012; 7: e43911.

30. Li DQ, Peng C, You HL, Lamanna MC, Harris JD, Lacovara KJ, et al. A Large Therizinosauroid (Dinosauria:Theropoda) from the Early Cretaceous of Northwestern China. Acta Geologica Sinica. 2007; 81: 539-49.

31. Zanno LE, Gillette DD, Albright LB, and Titus AL. A new North American therizinosaurid and the role of herbivory in 'predatory' dinosaur evolution. Proc Biol Sci, 2009; 276: 3505-11.

32. Xu X, Zhang XH, Sereno P, Zhao XJ, Kuang XW, Han J, et al. A new Therizinosauroid (Dinosauria, Theropoda) from the Upper Cretaceous iren Dabasu Formation of Nei Mongol. Vertebrata Palasiatica. 2002; 40: 228-40.

33. Barsbold R, Osmolska H, Watabe M, Currie PJ, and Tsogtbaatar K. A new oviraptorosaur (Dinosauria, Theropoda) from Mongolia: the first dinosaur with a pygostyle. Acta Palaeontologica Polonica. 2000; 45: 97-106.

34. Marshall AJ. Biology and comparative physiology of birds. Volume I. Quarterly Review of Biology. 1960; 2: iv.

35. Perle A, Chiappe LM, Barsbold R, Clark JM, and Norell MA. Skeletal Morphology of Mononykus olecranus (Theropoda: Avialae) from the Late Cretaceous of Mongolia. American Museum Novitates. 1994; 3105: 1-29.

36. Ostrom JH. Osteology of *Deinonychus antirrhopus*, an unusual theropod from the Lower Cretaceous of Montana. Peabody Museum of Natural History, Yale University Press. 1969; 30: 1-165.
37. Xu X, Han, FL, and Zhao Q. Homologies and homeotic transformation of the theropod ‘semilunate’ carpal. Scientific Report. 2014; 4, 6042. https://doi.org/10.1038/srep06042
38. Ji Q, and Ji SA. Protarchaeopterygid bird (*Protarchaeopteryx* gen. nov.)- fossil remains of archaeopterygids from China. Chinese Geology 1997; 238: 38-41
39. Gregory PS. The Segnosaurian Dinosaurs: Relics of the Prosauropod-Ornithischian Transition? Journal of Vertebrate Paleontology. 1984; 4: 507-15.
40. Osmólska H, Currie PJ, and Barsbold R. Oviraptorosauria. In: Weishampel DB, Dodson P, and Osmólska H (eds.), The Dinosauria (second edition). University of California Press, Berkeley. 2004; 7-19.
41. Ji Q, Norell MA, Makovicky PJ, Gao K-Q, Ji S, and Yuan C. An Early Ostrich Dinosaur and Implications for Ornithomimosaur Phylogeny. American Museum Novitates. 2003; 3420: 1-19.
42. Barsbold R. Opisthopubic pelvis in the carnivorous dinosaurs. Nature. 1979; 279: 792-93.
43. Norell MA, and Makovicky PJ. Important features of the dromaeosaur skeleton: information from a new specimen. American Museum Novitates.1997; 3215: 1-28.
44. Brochu CA. Osteology of *Tyrannosaurus rex*: Insights from a Nearly Complete Skeleton and High-Resolution Computed Tomographic Analysis of the Skull. Memoir Society of Vertevrate Paleontology. 2003; 22(sup4): 1-138.
45. Xu X, Wang XL, and Wu XC. A dromaeosaurid dinosaur with a filamentous integument from the Yixian Formation of China. Nature. 1999; 401: 262-66.

46. Xu X, Zhou ZH, and Wang XL. The smallest known non-avian theropod dinosaur. Nature. 2000; 408: 705.

47. Xu X, Zhou ZH, Wang XL, Kuang XW, Zhang FC, and Du XK. Four-winged dinosaurs from China. Nature. 2003; 421: 335-40.

48. Makovicky PJ, Apesteguia S, and Agnolin FL. The earliest dromaeosaurid theropod from South America. Nature. 2005; 437: 1007-11.

49. Xu X, Norell MA, Wang XL, Makovicky PJ, and Wu XC. A basal troodontid from the Early Cretaceous of China. Nature. 2002; 415: 780-4.

50. Forster CA, Sampson SD, Chiappe LM, and Krause DW. The theropod ancestry of birds: new evidence from the late cretaceous of Madagascar. Science. 1998; 279: 1915-19.

51. Perle A, Chiappe LM, Barsbold R, Clark JM, Norell MA. Skeletal morphology of *Mononychus olecranus* (Theropoda: Availae) from the Late Cretaceous of Mongolia. American Museum Novitates. 1994; 3105: 1–29.

52. Norell MA, and Makovicky PJ. *Dromaeosauridae*. In: Weishampel DB, Dodson P, and Osmólska H (eds.), The Dinosauria (second edition). University of California Press, Berkeley. 2004; 196-209.

53. Chiappe LM, Norrell MA, and Clark JM. Phylogenetic Position of *Mononykus* (Aves: Alvarezsauridae) from the Late Cretaceous of the Gobi Desert. Memoirs of the Queensland Museum. 1996; 39: 557-82.

54. Barsbold R, and Altangarel P. Segnosauria, a new infraorder of carnivorous dinosaurs. Acta Palaeontologica Polonica 1980; 25(2): 187-195

55. Kobayashi Y, and Lü JC. A new ornithomimid dinosaur with gregarious habits from the Late Cretaceous of China. Acta Palaeontologica Polonica. 2003; 48: 235-

56. Kobayashi Y, and Barsbold R. Ornithomimids from the Nemegt Formation of

Mongolia. Journal of Paleontological Society of Korea. 2006; 22: 195-207.

57. Lambe LM. The Cretaceous Theropodous Dinosaur *Gorgosaurus*. Canada Department of Mines and Geological Survey Memoir. 1917; 100: 1–84.

58. Lü JC, Pu HY, Kobayashi Y, Xu L, Chang HL, Shang YH, et al. A New Oviraptorid Dinosaur (Dinosauria: Oviraptorosauria) from the Late Cretaceous of Southern China and Its Paleobiogeographical Implications. Scientific Report. 2015; 5: 1-15.

59. Hou LH, Zhou ZH, Gu YC, and Zhang H. *Confuciusornis sanctus*, a new Late Jurassic sauriurine bird from China. Chinese Science Bulletin. 1995; 40: 1545.

Click here to access/download;Figure;Fig 1.tif

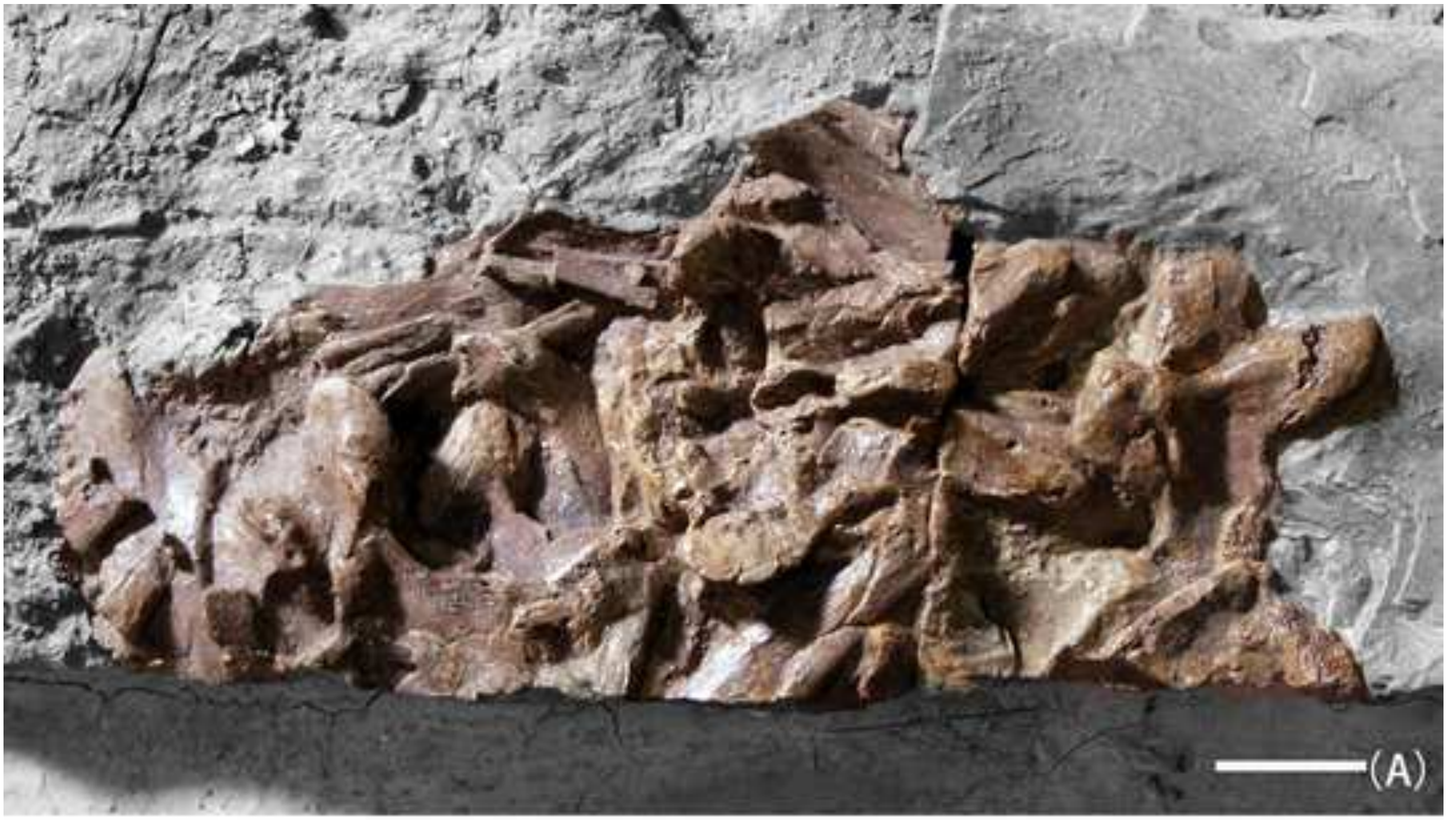

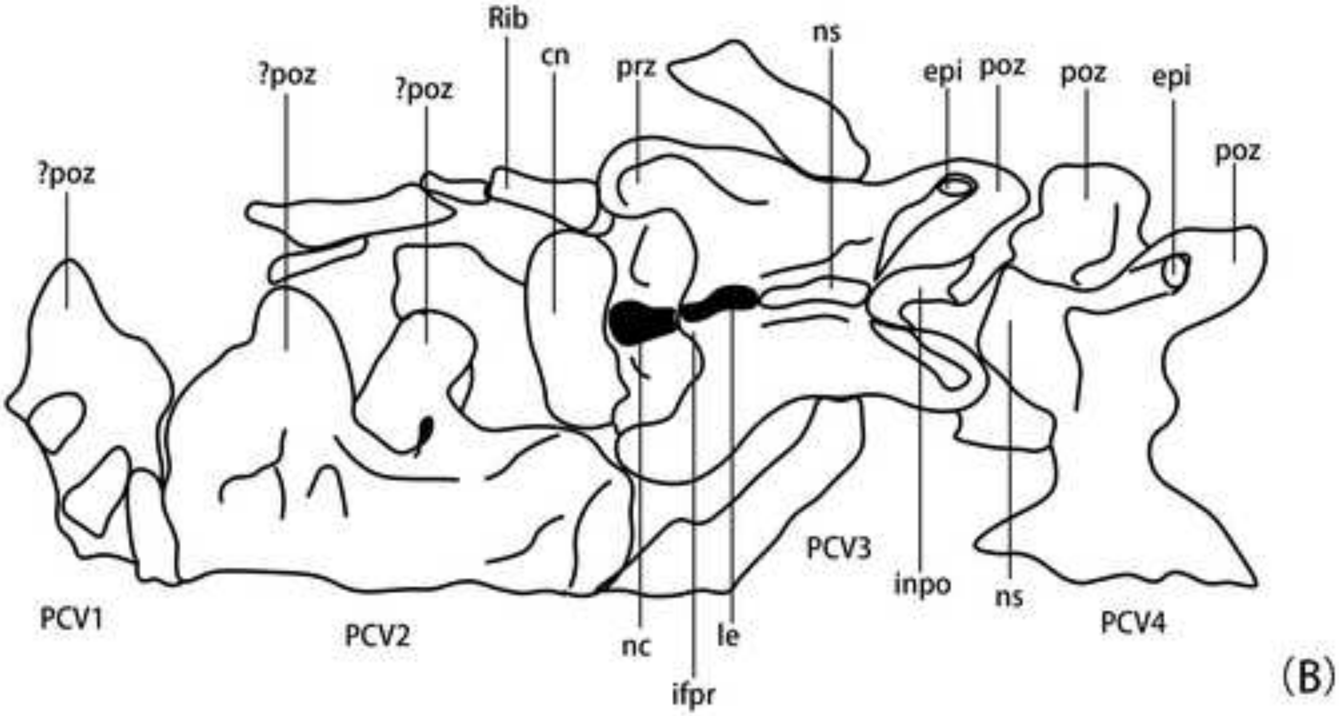

Click here to access/download;Figure;Fig 2.tif

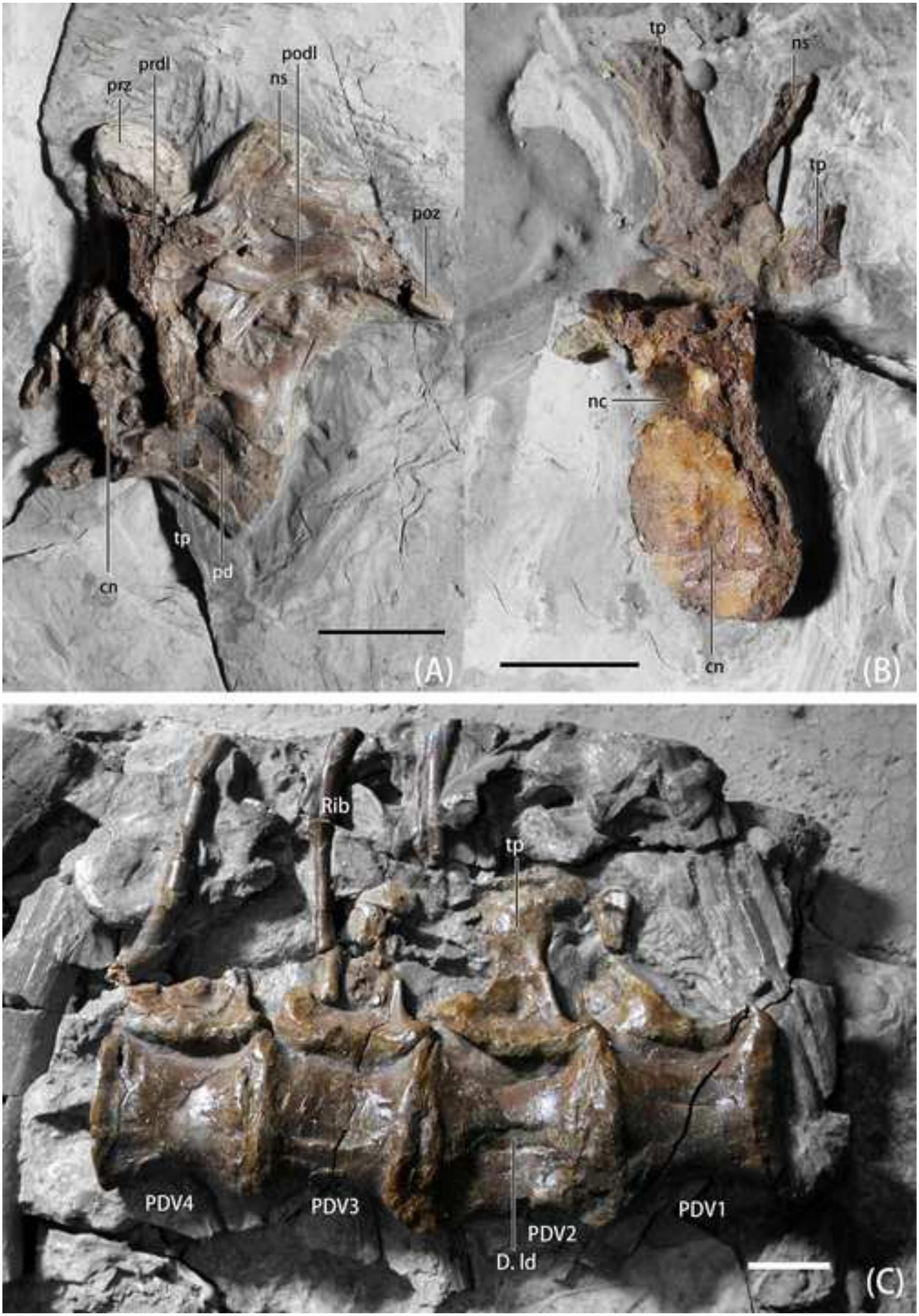

Click here to access/download;Figure;Fig 3 .tif

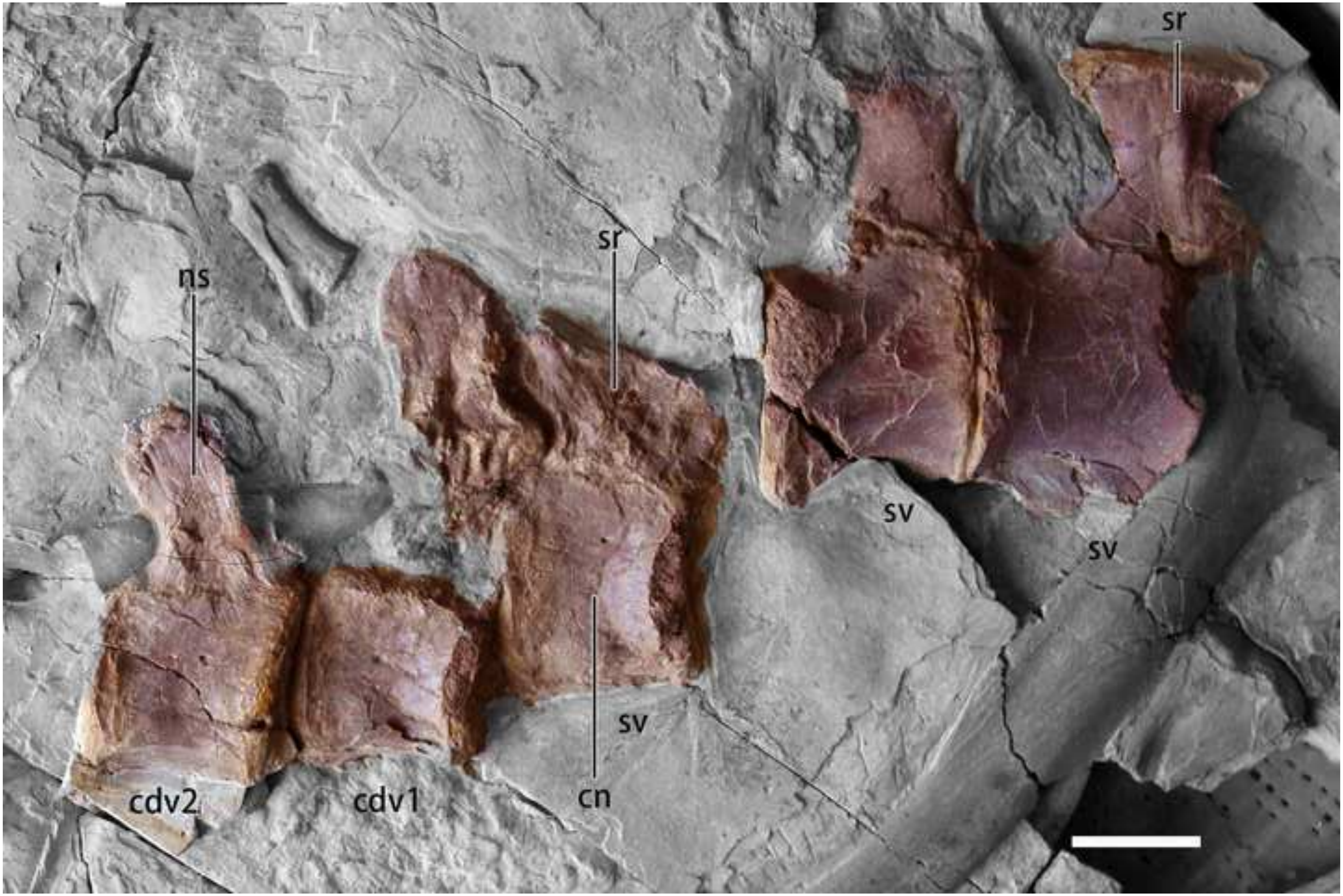

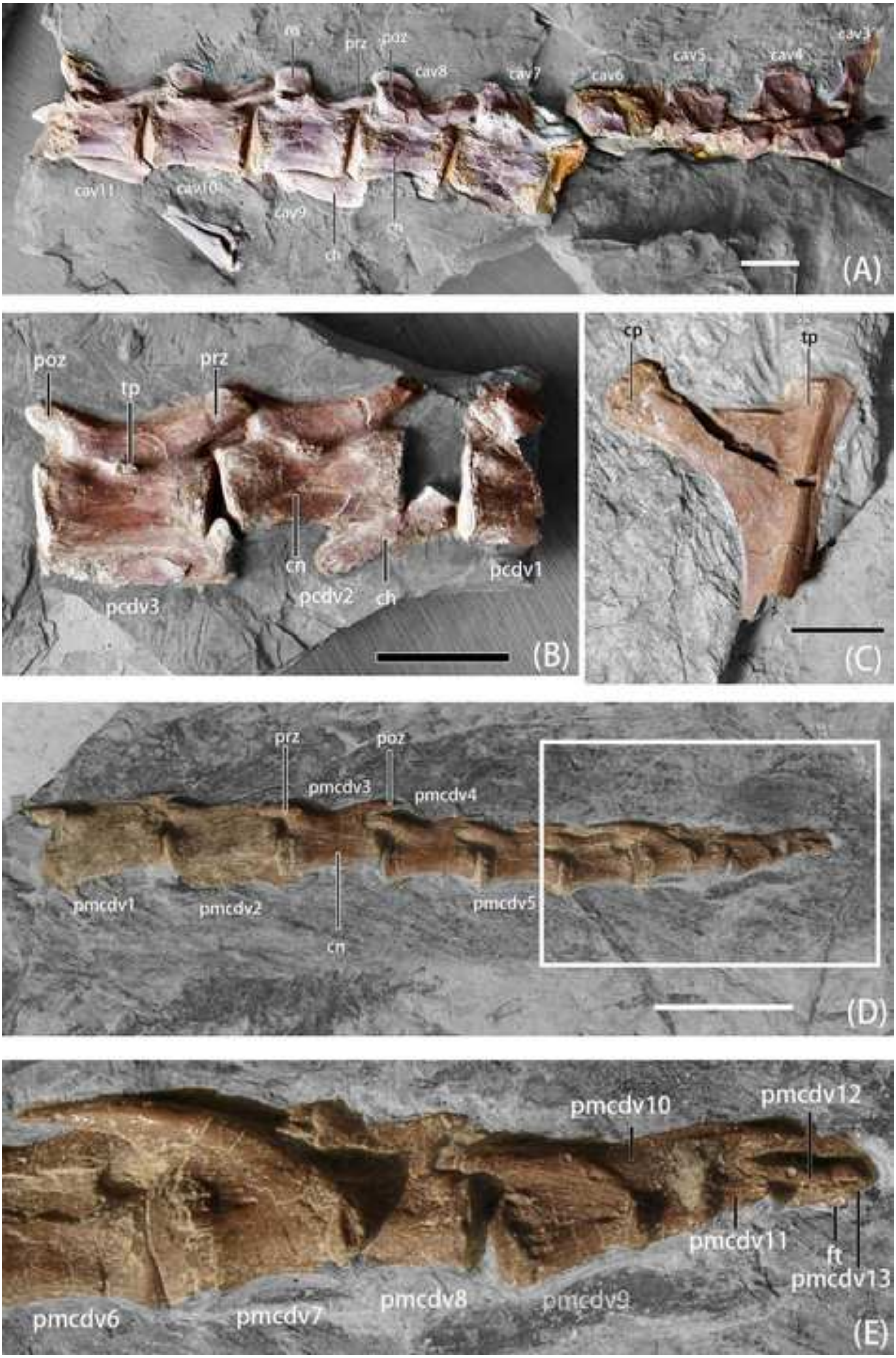

prz
poz
cav8
cav7
cav6
cav5
cav4
cav3
cav11
cav10
cav9
ch
(A)
poz
tp
prz
cn
pcdv1
pcdv2
pcdv3
ch
(B)
cp
tp
(C)
prz
poz
pmcdv3
pmcdv4
pmcdv1
pmcdv2
pmcdv5
cn
(D)
pmcdv10
pmcdv12
pmcdv11
ft
pmcdv13
pmcdv6
pmcdv7
pmcdv8
pmcdv9
(E)

Click here to access/download;Figure;Fig 5.tif

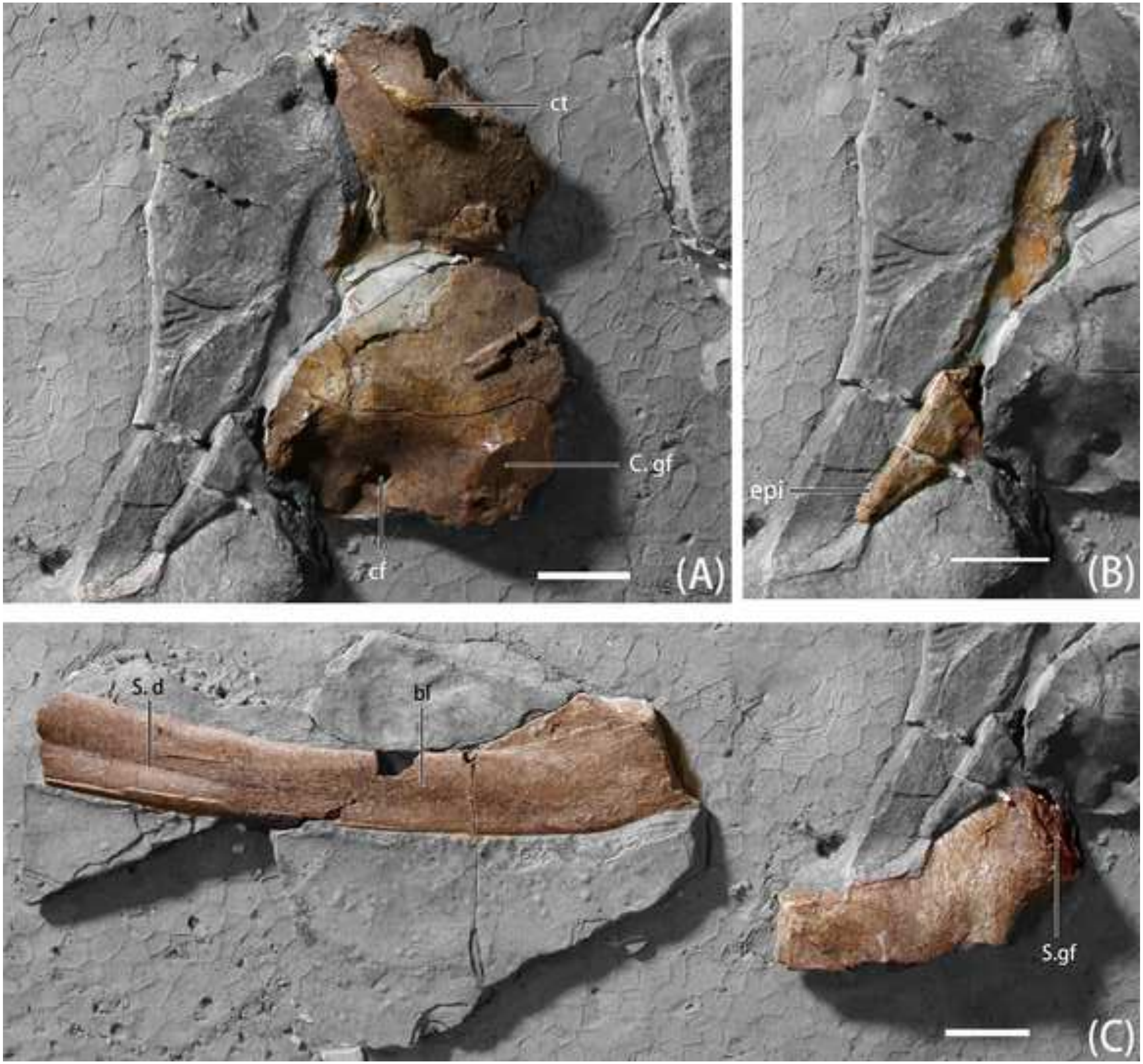

Click here to access/download;Figure;Fig 7.tif

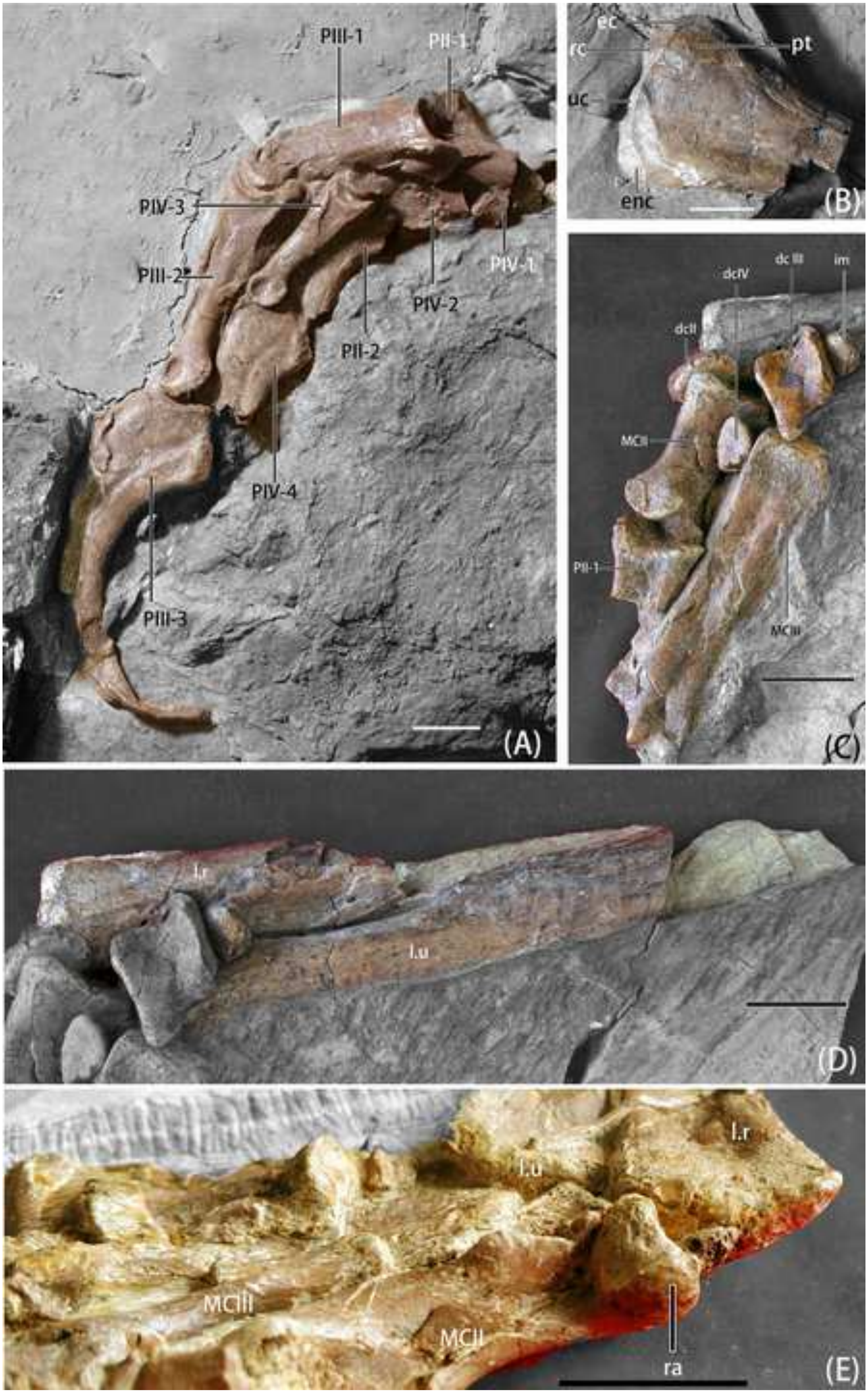

Click here to access/download;Figure;Fig 6.tif

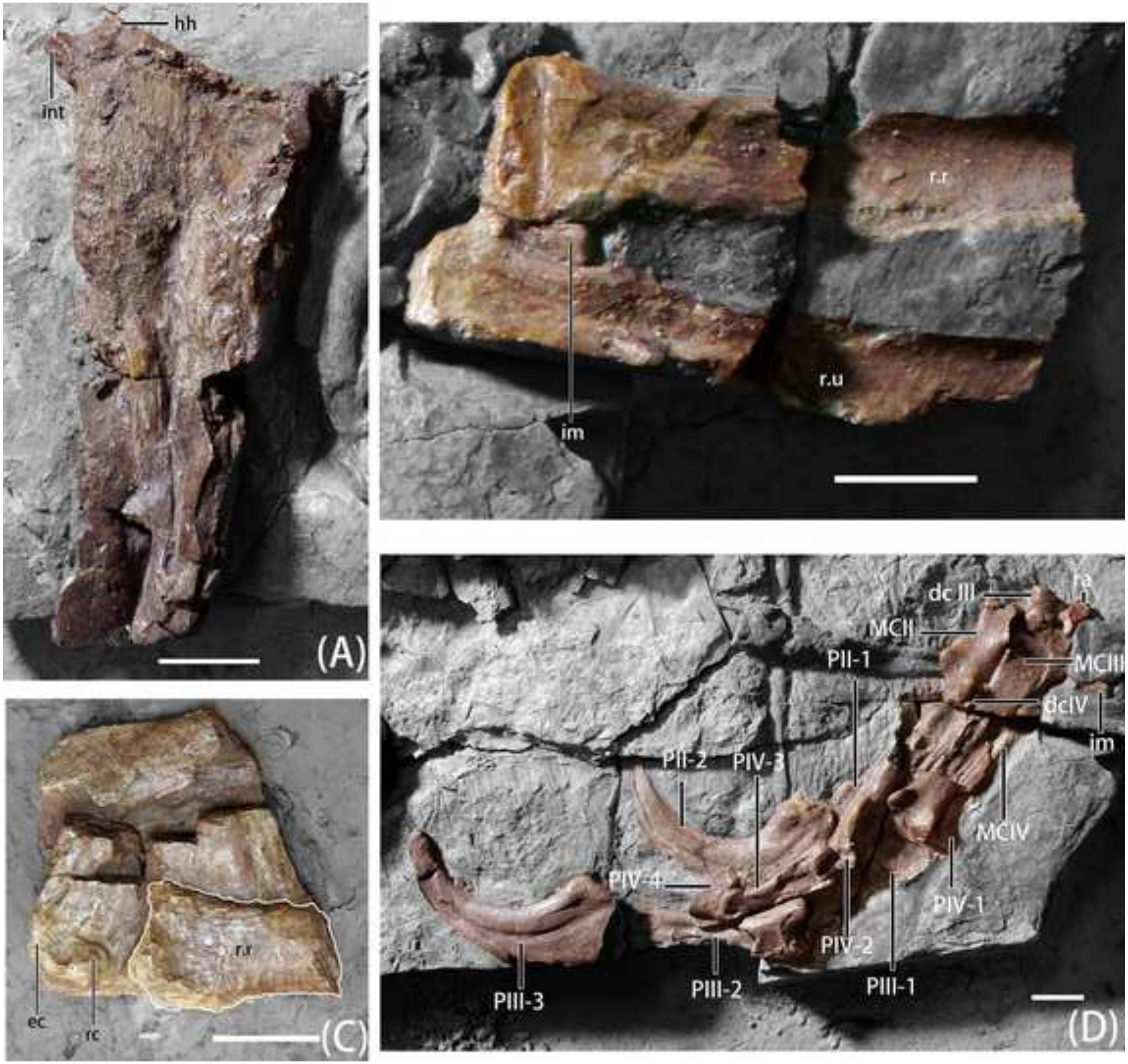

Click here to access/download;Figure;Fig 8.tif

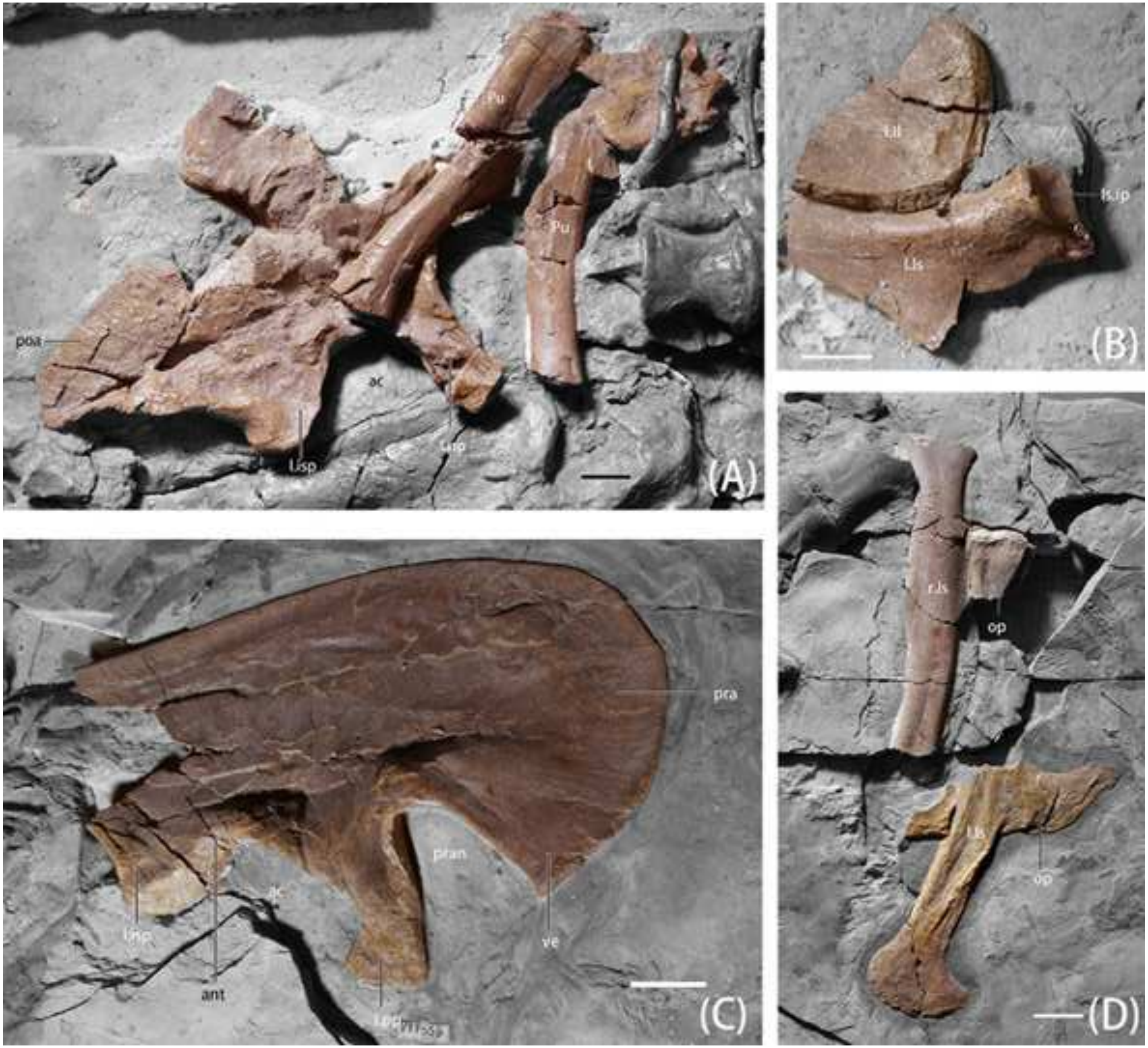

Click here to access/download;Figure;Fig 9.tif

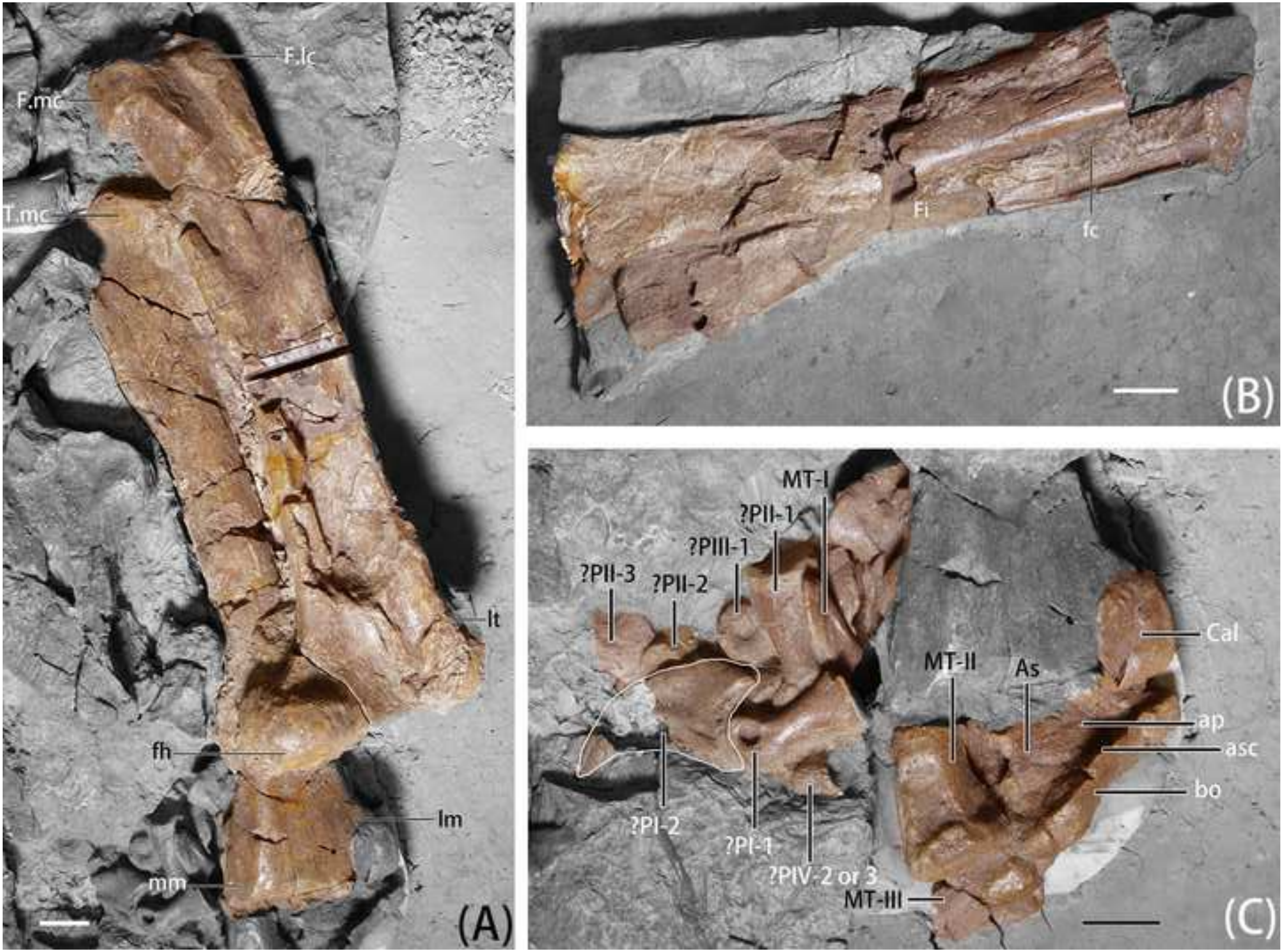

Supplement: Attachment — Submitted filename: PONE-D-21-14221_reviewer.pdf [file pone.0257913.s001.pdf]
