# Supplementary material for: Postcranial osteology of Beipiaosaurus inexpectus (Theropoda: Therizinosauria)
Source: PLoS One. 2021 Sep 30;16(9):e0257913. doi: 10.1371/journal.pone.0257913 (PMC8483305; doi:10.1371/journal.pone.0257913)
Supplement: Attachment — Submitted filename: Response to Reviewers.docx [file pone.0257913.s002.docx]

Key Laboratory of Vertebrate Evolution and Human Origins,

Institute of Vertebrate Paleontology and Paleoanthropology,

Chinese Academy of Sciences, Beijing 100044, China

[Jacky340@yahoo.com.tw](mailto:Jacky340@yahoo.com.tw)

16 July, 2021

Dear Dr. Joger,

We are thankful for your consideration of our manuscript and for the generous comments by the reviewers. We have updated the manuscript in light of the comments received, and believe that our revised submission is now suitable for publication.

Below, we enumerate the changes we have made to the manuscript in response to the requested revisions:

1) **In journal requirements, the permits are asked to state in manuscript.**

The specimen has been published before and housed in IVPP, so no field or related permits are needed in this study. We have added the statement in Methods (Lines 79-80).

2) **Journal requirements indicates “We note that Figure(s) 1, 2, 3, 4, 5, 6, 7, 8 and 9 in your submission contain copyrighted images…”.**

All the figures were drawn or token by authors of this article, so all of them are not under copyright. We have made a more detailed statement in “Copyrighted Figures” of “Additional Information”.

3) **Reviewer 1 suggests explaining the reason of terminology choosing of manual digits (Lines 73-75).**

The choosing of the terminology is to in light of the recent research of avian digit homology. We have added the reason in lines 75-76.

4) **Reviewer 1 suggests using only genetic name or genetic and species name in lines 130-135.**

We fixed it into genetic name only throughout the paragraph, and note the species name and number when referring to specific specimen.

5) **Reviewer 1 suggests a more detailed description in line 328 instead of just saying “different”.**

We have added a brief description of the chevrons of *Falcarius* and *Alxasaurus* to show how do they different from the chevrons of *Beipiaosaurus* (Lines 328-329).

6) **Both reviewers found the sentence in lines 342-345 needs rewording.**

We have miswritten the sentence and reworded it to make the contents more reasonable now (Lines 342-343).

Many thanks for your consideration, and best wishes,

Chun-Chi Liao

On behalf of all authors.
